# 2017 publication guidelines for structural modelling of small-angle scattering data from biomolecules in solution: an update

**DOI:** 10.1107/S2059798317011597

**Published:** 2017-08-18

**Authors:** Jill Trewhella, Anthony P. Duff, Dominique Durand, Frank Gabel, J. Mitchell Guss, Wayne A. Hendrickson, Greg L. Hura, David A. Jacques, Nigel M. Kirby, Ann H. Kwan, Javier Pérez, Lois Pollack, Timothy M. Ryan, Andrej Sali, Dina Schneidman-Duhovny, Torsten Schwede, Dmitri I. Svergun, Masaaki Sugiyama, John A. Tainer, Patrice Vachette, John Westbrook, Andrew E. Whitten

**Affiliations:** a School of Life and Environmental Sciences, The University of Sydney, NSW 2006, Australia; b ANSTO, New Illawarra Road, Lucas Heights, NSW 2234, Australia; c Institut de Biologie Intégrative de la Cellule, UMR 9198, Bâtiment 430, Université Paris-Sud, 91405 Orsay CEDEX, France; d Université Grenoble Alpes, Commissariat à l’Energie Atomique (CEA), Centre National de la Recherche Scientifique (CNRS), Institut de Biologie Structurale (IBS), and Institut Laue–Langevin, 71 Avenue des Martyrs, 38000 Grenoble, France; e Department of Biochemistry and Molecular Biophysics, Columbia University, New York, NY 10032, USA; f Molecular Biophysics and Integrated Bioimaging, Lawrence Berkeley National Laboratory, Berkeley, CA 94720, USA; g University of Technology Sydney, ithree Institute, 15 Broadway, Ultimo, NSW 2007, Australia; h Australian Synchrotron, 800 Blackburn Road, Clayton, VIC 3168, Australia; i Synchrotron SOLEIL, L’Orme des Merisiers, Saint-Aubin BP48, 91192 Gif-sur-Yvette CEDEX, France; j School of Applied and Engineering Physics, Cornell University, Ithaca, NY 14853-2501, USA; k Department of Bioengineering and Therapeutic Sciences, Department of Pharmaceutical Chemistry, and California Institute for Quantitative Biosciences (QB3), University of California San Francisco, San Francisco, California, USA; l School of Computer Science and Engineering, Institute of Life Sciences, The Hebrew University of Jerusalem, Jerusalem 9190401, Israel; m Biozentrum, University of Basel and SIB Swiss Institute of Bioinformatics, Basel, Switzerland; n European Molecular Biology Laboratory (EMBL) Hamburg, c/o DESY, Nokestrasse 85, 22607 Hamburg, Germany; o Research Reactor Institute, Kyoto University, Kumatori, Sennan-gun, Osaka 590-0494, Japan; p Basic Science Research Division, Molecular and Cellular Oncology, MD Anderson Cancer Center, University of Texas, Houston, Texas, USA; q Department of Chemistry and Chemical Biology, Rutgers University, New Brunswick, NJ 07102, USA

**Keywords:** small-angle scattering, SAXS, SANS, biomolecular structure, proteins, DNA, RNA, structural modelling, hybrid structural modelling, publication guidelines, integrative structural biology

## Abstract

In 2012, preliminary guidelines were published addressing sample quality, data acquisition and reduction, presentation of scattering data and validation, and modelling for biomolecular small-angle scattering (SAS) experiments. Bio­molecular SAS has since continued to grow and authors have increasingly adopted the preliminary guidelines. In parallel, integrative/hybrid determination of biomolecular structures is a rapidly growing field that is expanding the scope of structural biology. For SAS to contribute maximally to this field, it is essential to ensure open access to the information required for evaluation of the quality of SAS samples and data, as well as the validity of SAS-based structural models. To this end, the preliminary guidelines for data presentation in a publication are reviewed and updated, and the deposition of data and associated models in a public archive is recommended. These guidelines and recommendations have been prepared in consultation with the members of the International Union of Crystallography (IUCr) Small-Angle Scattering and Journals Commissions, the Worldwide Protein Data Bank (wwPDB) Small-Angle Scattering Validation Task Force and additional experts in the field.

## Abstract 2

Updated guidelines are presented for publishing biomolecular small-angle scattering (SAS) experiments so that readers can independently assess the quality of the data and models presented. The focus is on solution scattering experiments with either X-rays (SAXS) or neutrons (SANS), where the primary goal is the generation and testing of three-dimensional models, particularly in the context of integrative/hybrid structural modelling.

## 1. Introduction   

The objective of publishing the preliminary guidelines for biomolecular small-angle scattering (SAS) experiments (Jacques, Guss, Svergun *et al.*, 2012 ▸; Jacques, Guss & Trewhella, 2012 ▸) was to provide a reporting framework so that ‘readers can independently assess the quality of the data and the basis for any interpretations presented’. The focus was on solution SAS experiments, both small-angle X-ray scattering (SAXS) and small-angle neutron scattering (SANS), where the primary goal is the generation and testing of three-dimensional models. The 2012 guidelines, which were developed in consultation with members of the SAS and Journals Commissions of the IUCr and other experts in the field, are now used by many authors and are endorsed by IUCr Journals (http://journals.iucr.org/services/sas/).

Since the preliminary publications appeared, the Worldwide Protein Data Bank (wwPDB) established the Small-Angle Scattering Validation Task Force (SASvtf; https://www.wwpdb.org/task/sas), which has made recommendations regarding the archiving and validation of SAS data and models (Trewhella *et al.*, 2013 ▸). Furthermore, the wwPDB Integrative/Hybrid Methods (IHM) Validation Task Force was formed (Sali *et al.*, 2015 ▸) to address the complex issues concerning the archiving and validation of models of bio­molecular complexes and assemblies that depend upon computational methods and data from independent experimental techniques, including SAS. There also have been substantial advances in analysis tools for SAS (Franke *et al.*, 2015 ▸; Rambo & Tainer, 2013*b* ▸; Schneidman-Duhovny *et al.*, 2013 ▸; Petoukhov & Svergun, 2015 ▸; Konarev & Svergun, 2015 ▸; Petoukhov *et al.*, 2012 ▸; Chen & Hub, 2015 ▸; Spinozzi *et al.*, 2014 ▸; Bizien *et al.*, 2016 ▸) and instrumentation, in particular the growth of SAS experiments utilizing inline purification and characterization (Blanchet *et al.*, 2015 ▸; Jordan *et al.*, 2016 ▸; Graewert *et al.*, 2015 ▸; Brookes *et al.*, 2013 ▸, 2016 ▸; Bras *et al.*, 2014 ▸; Meisburger *et al.*, 2016 ▸; Ibrahim *et al.*, 2017 ▸). In regard to modelling SAS data, there has been significant increased interest and methods development in multistate/ensemble-based methods for flexible biomolecules (Tria *et al.*, 2015 ▸; Berlin *et al.*, 2013 ▸; Schneidman-Duhovny *et al.*, 2016 ▸; Perkins *et al.*, 2016 ▸; Kikhney & Svergun, 2015 ▸; Terakawa *et al.*, 2014 ▸) and structural modelling based on combined SAS and NMR data (Schwieters & Clore, 2014 ▸). The latter places especially stringent requirements on the accuracy and precision of SAS data.

The recommendations of the SASvtf (Trewhella *et al.*, 2013 ▸) have progressed substantially with regard to model validation and archiving. Work also has begun on the community discussions and technical developments required to develop a federated system of data banks to support the dissemination and validation of integrative/hybrid models (Sali *et al.*, 2015 ▸). In particular:

(i) a standard dictionary with definitions of terms for collecting and managing SAS data as well as facilitating data exchange between laboratories and data banks has been developed (Kachala *et al.*, 2016 ▸), building upon the sasCIF (Malfois & Svergun, 2000 ▸) that was originally developed as an extension of the core Crystallographic Information Framework (CIF);
(ii) a freely accessible and fully searchable SAS experimental data and model data bank (SASBDB; https://www.sasbdb.org/; Valentini *et al.*, 2015 ▸) has been established to be part of an envisioned federated system of interoperable data banks supporting hybrid data and model validation.

The SASvtf report reiterated the importance of the recommended preliminary publication guidelines and expanded on them, further stating that ‘criteria need to be agreed upon for the assessment of the quality of deposited data, the accuracy of SAS-derived models, and the extent to which a given model fits the SAS data’.

In the light of the above developments, it is timely to update the preliminary publication guidelines. We have followed the same structure as previously, with four sections covering (i) sample quality, (ii) data acquisition and reduction, (iii) the presentation of scattering data and validation, and (iv) structure modelling. Each section briefly describes the relevant context with a tabulated summary of the specific information to be reported. Importantly, we have added a recommendation that SAS data and models, along with the details of the experiment as described in each of the four sections here, be deposited in a public data bank. An example report is provided at the end of these sections for a specific set of size-exclusion chromatography SAXS (SEC–SAXS) experiments in a form that is consistent with the guidelines and demonstrates the value of complete reporting. While many of the recommended guidelines are best practice for biomolecular SAS generally, our main focus remains on experiments aimed at three-dimensional structural modelling from solution SAS data. As such, SAS experiments aimed at understanding highly heterogeneous mixtures, transient species using time-resolved data, or high-throughput screening experiments are not explicitly considered as each of these important applications would have distinct attributes that need to be considered separately in detail.

Importantly, the guidelines are not intended to restrict publication, but rather to ensure adequate description of the accuracy and confidence in the data and modelling outputs. The objective is to ensure that the reader understands the accuracy and precision of the derived parameters and models and any limitations to the data. This understanding is essential for quantifying uncertainty in IHM structural modelling using SAS data (Schneidman-Duhovny *et al.*, 2014 ▸; Yang *et al.*, 2012 ▸). It is also important in evaluating data that might be limited in some way and yet still provide reliable structural insights.

## 2. Context for the guidelines   

### 2.1. Sample quality   

Given the paramount importance of sample preparation and characterization for biomolecular structure modelling using SAS data, sample quality must continue to be emphasized. A SAS profile can be measured from any sample and, unlike crystallography and NMR where there are both quantitative standards and internal controls for assessing sample and data quality, a SAS profile by itself does not provide sufficient information for such assessment. Fundamental to the successful interpretation of a biomolecular SAS experiment in terms of structural models is that the scattering data are demonstrated to be from a highly purified solution of monodisperse particles in the dilute solution regime. This means that the SAS data are free of contributions from contaminants and the effects of nonspecific aggregation or inter-particle distance correlations. To avoid these systematic biases, well characterized solutions of high purity must be measured, yielding SAS profiles that encode information regarding biomolecular structure (the form factor). Additionally, as coherent scattering that encodes the desired structural information for a biomolecule in solution is inherently weak (*e.g.* ∼1 in 10^6^ incident photons are scattered from a 1 mg ml^−1^ solution of a 15 kDa protein; Stuhrmann, 1980 ▸), accurately and precisely scaled measurements, with respect to incident radiation, of solvent plus biomolecule and precisely matched solvents also are essential. As described in the following sections, an inaccurate solvent subtraction from the solvent plus the biomolecule of interest will affect important validation parameters and structural interpretation.

Traditionally, solution SAS data for structural evaluation and modelling have been collected at multiple concentrations of the particle of interest to evaluate and eliminate concentration-dependent contributions to the scattering through the strategic choice of solvent conditions or extrapolation to infinite dilution. The molecular mass (*M*) or volume (*V*) of the scattering particle then can be estimated from the zero-angle scattering, *I*(0). The calculation of *M* or *V* from *I*(0) requires accurate concentrations of the sample constituents to be determined, which can be challenging. While UV-based determination of concentration can be difficult for some systems (for example proteins with few aromatics or with solvents containing UV-absorbing components), concentration can often be determined to better than 10% accuracy (Gasteiger *et al.*, 2005 ▸). Agreement of the *I*(0)-based estimate of *M* with that determined from the chemical composition of the scattering particle is important in validating that the measured SAS profile corresponds to the form factor of the particle of interest, is free of nonspecific associations and is in the dilute solution regime. When determining *M* from chemical composition it is important to include not only the protein or nucleic acid sequence, but also purification tags if still present, plus any cofactors, modifications or bound ligands, and in the case of SANS the isotopic composition. There may be situations where the determination of *M* from *I*(0) differs from that calculated from the composition. For example, DNA and RNA as polyanions can attract a diffuse ion atmosphere where neutralizing counterions are localized near their surface and will contribute significantly to the scattering. These effects on particle scattering can be difficult to quantify *a priori*. In such cases, there should be some discussion dedicated to explaining any major discrepancies from the expected *M*.

In the case of folded structures, and providing that solvent subtraction is accurate, one can use the complementary method for estimating *M* using the scattering invariant (*Q_i_*; Porod, 1951 ▸) and its relationship to the scattering particle volume (Debye *et al.*, 1957 ▸; Porod, 1951 ▸). In the case of unfolded or very flexible systems, the Kratky plot (Kratky, 1982 ▸) can provide evidence for the flexibility. Solvent-blank mismatch with the sample will introduce errors that will confound these analyses as they depend on an accurate representation of the scattering at high angles. For proteins, the high-angle data are orders of magnitude less intense than the lowest angle data, and are only a few parts per thousand above the solvent scattering. For SANS data, contributions to the background from incoherent scattering can also prove problematic as the incoherent scattering cross-section of ^1^H is 10–20 times the total scattering cross-sections of other nuclei present in a biomolecule (Jacrot, 1976 ▸). As a result, solvent subtractions for SANS data with significant ^1^H often include adjustments by an *ad hoc* addition or subtraction of a constant to force the scattering at high angles to approximately zero. The need for this adjustment can be minimized by using a final dialysate as the solvent blank from dialysis that has been maintained in a closed environment to avoid differential ^1^H–^2^H exchange and calibrating sample and solvent transmissions against pure ^1^H_2_O and ^2^H_2_O.

Developments of inline purification of samples using size-exclusion chromatography (SEC) at synchrotron SAXS beamlines (see, for example, Brennich *et al.*, 2017 ▸; David & Pérez, 2009 ▸; Graewert *et al.*, 2015 ▸; Mathew *et al.*, 2004 ▸) and at SANS beamlines (Jordan *et al.*, 2016 ▸) is becoming increasingly popular. These SEC–SAS measurements involve the collection of SAS data as the solution elutes from the SEC column, and thus enable the separation of components of mixtures and polydisperse solutions. In the case of membrane proteins, this allows the separation of encapsulated proteins from empty detergent micelles or nanodiscs (Berthaud *et al.*, 2012 ▸). This combined SEC–SAS approach has been extremely successful at synchrotron SAXS facilities and has opened up studies of systems that were previously impossible owing to time-dependent aggregation. A drawback to the approach is the necessary dilution of the sample on the SEC column. Additionally, as the fluid in the centre of the tubing linking the SEC column to the SAXS cell flows faster than that at the edges of the tube (Poiseuille flow), the SEC peak will broaden before measurement. Depending on the path length between the measurement cell/capillary and the end of the column, this broadening can be quite significant. Excessive path lengths will not only degrade the resolution of the eluted peaks, but the UV-absorbance measurements of the eluent may not correlate with the SAS measurement frame, which limits the ability to determine sample concentrations. Monitoring UV absorbance immediately prior to SAS measurements with minimal intervening path length and volume, or ultimately with coincident measurement, facilitates increased accuracy in the estimation of *M* or *V* from *I*(0).

Excellent descriptions for the preparation of high-quality samples and well matched solvent blanks for SAXS and SANS experiments have recently appeared in *Nature Protocols* (Jeffries *et al.*, 2016 ▸; Skou *et al.*, 2014 ▸). Together, these papers provide important and comprehensive practical advice for the preparation of samples for a SAS experiment that demonstrably meet the stringent requirements for obtaining SAS data suitable for structural analysis. Table 1 ▸ summarizes our recommended reporting guidelines for sample details.

### 2.2. Data acquisition and reduction   

In the case of isotropic solution scattering, data reduction refers to the process of converting counts on a detector to the one-dimensional scattered intensity profile arising from the sample, with associated errors, as *I*(*q*) *versus q* (where *q* = 4πsinθ/λ, 2θ is the scattering angle and λ is the wavelength of the radiation). To obtain the SAS profile relating to the structure of the particle of interest, the data-reduction software must take into account detector sensitivity and non­linearity, sample transmission, incident intensity and accurate and precise subtraction of solvent scattering. Dilute solution measurement places severe requirements on normalizing scattering intensity measurements, which today can be better than 0.1% and fully satisfactory. All of these procedures are described in detail in Svergun *et al.* (2013 ▸).

The data-reduction process may also require addressing potential instrumental ‘smearing’ effects on the SAS profile (see chapter 4 of Glatter & Kratky, 1982 ▸). The theory guiding the interpretation of SAS data in terms of structure generally assumes an effective point source and a single wavelength. The instrument setup used for a SAS experiment may be an excellent approximation to a point source, or may differ significantly from it and thus require corrections to be made to data or to model scattering profiles for comparison with the experiment. The wavelength resolution (Δλ/λ) for SAXS (whether synchrotron or laboratory-based) is generally a good approximation to a single wavelength, while for SANS it can be of the order of 10–15% in order to optimize the neutron flux on the sample (for examples, see https://www.ill.eu/instruments-support/instruments-groups/groups/lss/more/world-directory-of-sans-instruments/). Beam size and shape also play a key role in data smearing. Modern synchrotron beams and most laboratory-based instruments have sufficiently small beam dimensions (in the range of tenths of a millimetre to millimetres at the detector) such that smearing effects can be safely ignored for most applications. Neutron beam dimensions can be as large as 100 mm at the detector and thus can cause significant instrumental smearing. Some laboratory-based SAXS instruments use line-focused sources to increase the X-ray flux on the sample. These types of instruments, which were first implemented by Otto Kratky (see chapter 3 of Glatter & Kratky, 1982 ▸), have since been further developed for laboratory-based SAS applications (see, for example, Bergmann *et al.*, 2000 ▸) and data treatments must deal with significant instrumental smearing effects. Data ‘desmearing’ can be performed using the ratio of points in the smeared-model and unsmeared-model *I*(*q*) profiles calculated using Fourier and/or linear regularization techniques, such as the indirect Fourier transform of a *P*(*r*) model if the particle maximum dimension (*d*_max_) is well determined. Alternatively, iterative methods can be used, although these typically amplify statistical errors (see Vad & Sager, 2011 ▸ and references therein). However, the preferred approach is to smear the model *I*(*q*) profile analytically using the measured beam profile for direct comparison with experimental data.

During data reduction, the SAS intensity data also should be placed on an absolute scale in units of cm^−1^ by comparison with the incident beam flux or the scattering from pure H_2_O (Orthaber *et al.*, 2000 ▸; Jacrot & Zaccai, 1981 ▸). Pure H_2_O is a readily accessible, universal standard whose scattering has been well characterized over a wide range of temperatures. Secondary standards are also available, such as glassy carbon (see the new NIST Standard Reference Material 3600; https://www-s.nist.gov/srmors/view_detail.cfm?srm=3600; Allen *et al.*, 2017 ▸). Absolute scaling enables the direct comparison of SAS data from different instruments, including X-ray and neutron sources, without arbitrary scaling and also enables the determination of *M* or *V* from *I*(0) without reference to the scattering from a reference protein. In the case of SANS, it has been routine to place the data on an absolute scale. The more common practice for SAXS experimenters has been to provide data on an arbitrary relative scale, which we do not recommend for reasons that will be addressed further below.

Owing to the tremendous variety of SAS instrumentation, the typical SAS user will need beamline scientists or instrument manufacturers to provide many of the instrument and data-acquisition parameters and references that we recommend to be reported regarding data acquisition and reduction (a summary is given in Table 2 ▸). We therefore encourage instrument scientists to collect and provide these parameters and references to users in an easy-to-access form at the time of data collection.

### 2.3. Data presentation, analysis and validation   

In order for a reader to be able to assess the quality of SAS data and their suitability for structural modelling, it is necessary that the data be presented in a clear, well described manner along with the parameters and analyses that support the conclusion that the SAS profile represents the shape of the particle of interest or, in the case of flexible systems, the population-weighted average SAS profile for the ensemble of conformations present.

Because *I*(*q*) decreases by several orders of magnitude over the measured *q* range, data should be presented as log *I*(*q*) *versus* *q* and/or log *I*(*q*) *versus* log *q*. The former provides a clear representation of the data over the entire *q* range, while the latter will have a near-zero slope at low *q* if the minimum measured *q* value meets the requirement of being sufficiently small to ensure adequate characterization of the largest particles present. A linear Guinier plot [ln *I*(*q*) *versus* *q* ^2^; Guinier, 1939 ▸] is a necessary but not sufficient demonstration that a solution contains monodisperse particles of the same size. The upper limit of the *q* range for the linear Guinier approximation varies depending on the particle shape and homogeneity. For a sphere of uniform scattering density, Guinier showed that the limit is *qR*_g_ < 1.3, while for extended shapes and/or inhomogeneous particles this limit can be <1.0 (Feigin & Svergun, 1987 ▸). Assessment of the appropriate Guinier limit will be aided by complementary analyses for particle shape, such as *P*(*r*) (see below). The lower *q* limit for the Guinier analysis should be the lowest, reliably measured *q* value. For a particle with maximum dimensions *d*_max_, the minimum *q* value measured should be at most ∼π/*d*_max_ for accurate assessment of the particle size and shape (Moore, 1980 ▸), and as a general principle it is important to measure below this limit to have an assurance that there are no larger particles present. It has been common practice to truncate data at low *q* when there are small amounts of large *M* impurities, aggregation or polydispersity present resulting in some upturn of the Guinier plot. This practice is not to be encouraged, but in the event that it is performed it must be reported and justified. Truncating the most obviously affected lower *q* data in the Guinier plot will not completely eliminate the effects of the contaminant and will thus have an effect on the derived structural parameters that must be acknowledged and quantified to the extent possible [for example, by indicating the impacts on *I*(0) and *R*_g_]. The best practice would be to also display the truncated data points, for example as empty symbols, with filled symbols representing data points included in the linear fit so that the reader can fully appreciate the potential effect of truncation. For Guinier fits, a quality-of-fit parameter such as the Pearson residual (*R*) or coefficient of correlation (*R* ^2^) for a linear fit is widely understood and thus is most useful to report.

The Fourier transform of the scattering profile yields *P*(*r*) *versus* *r*, the scattering contrast-weighted distribution of distances *r* between atoms, and is generally computed as the indirect Fourier transform of *I*(*q*) (Glatter, 1977 ▸). By its definition, *P*(*r*) is equal to zero for *r* values exceeding the maximum particle size *d*_max_. Agreement between the *P*(*r*) and Guinier-determined *R*_g_ and *I*(0) values is a good measure of the self-consistency of the SAS profile, as *P*(*r*) is calculated using a larger portion of the measured *q* range. This said, it is not correct to simply choose a *d*_max_ that provides a solution that agrees with the Guinier *R*_g_. Rather, the *P*(*r*) solution must be independently optimized with the understanding that *d*_max_ is an input parameter to the indirect transform selected by the user based on the observed fit of the regularized *I*(*q*) corresponding to a given *P*(*r*) and how *P*(*r*) approaches zero at *r* = 0 and *d*_max_. The *d*_max_ value as independently assessed from the *P*(*r*) transform should be consistent with, but not guided by, the known dimensions of the system from complementary techniques. There is an inherent uncertainty in *d*_max_ that is difficult to quantify in a rigorous and consistent way. Furthermore, automated routines for calculating *P*(*r*) can provide mathematically optimized solutions that are quite unphysical, leading to erroneous *d*_max_ selection, and hence need to be treated with great caution. The stability of the *P*(*r*) fit needs to be carefully assessed by examining a range of *d*_max_ values and the effects of choosing different *q* ranges. The indirect Fourier transform methods for calculating *P*(*r*) include a smoothing parameter that is a complicating factor in assessing the quality of the fit for a given solution. A simple *χ* ^2^ test is straightforward to calculate, although it does have limitations, as will be discussed below (§[Sec sec2.4]2.4). Another approach used by the popular program *GNOM* for calculating *P*(*r*) is to use a quality-of-fit assessment (referred to as the ‘total estimate’ ) that is based on χ^2^ combined with a number of ‘perceptual criteria’ (Svergun, 1992 ▸).

The molecular mass *M* in daltons for a scattering particle is readily calculated as
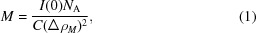
where *I*(0) is on an absolute scale in units of cm^−1^, *N*_A_ is Avogadro’s number, *C* is the concentration of the scattering particle in g ml^−1^ and Δρ_*M*_ is the scattering mass contrast, which can be calculated as 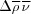, where 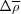 is the average scattering-length density difference between the particle and its solvent in cm^−2^ (or cm cm^−3^, scattering length/unit volume) and 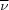 is its partial specific volume in cm^3^ g^−1^ (Orthaber *et al.*, 2000 ▸). 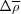 and 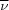 are both related to the molecular volume and can be readily estimated for X-rays and neutrons from the chemical and isotopic composition of the particle and its solvent. For X-rays, Δρ_*M*_ is sometimes calculated as 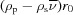, where ρ_p_ is the number of electrons per mass of dry volume, ρ_s_ is the electron density of the solvent and *r*_0_ is the scattering length of an electron in cm (2.8179 × 10^−13^ cm; Mylonas & Svergun, 2007 ▸). There are several web-based tools for the calculation of these parameters from the chemical and isotopic composition. Values for 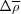 and 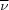 from the chemical composition of solvent and solute for SAXS and SANS can be obtained using the Contrast model of *MULCh* (http://smb-research.smb.usyd.edu.au/NCVWeb/index.jsp); the web version of *US-SOMO* (https://somo.chem.utk.edu/somo/) will calculate 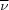 and other molecular properties from the sequence. A biomolecular scattering-length density (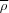) calculator for proteins and polynucleotides with different levels of deuteration is also available at http://psldc.isis.rl.ac.uk/Psldc/. These calculations are based on the volumes of the constituent chemical groups and generally provide accurate values of 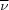 for proteins with *M* > 20 kDa, where the effects of hydration and variations in amino-acid packing have little impact on calculations. For an easy-to-use protocol for the calculation of *M*, see Box 2 in Jeffries *et al.* (2016 ▸).

Historically, proteins have been used as a calibration standard for estimating *M*. From (1) it can be seen that if the product of 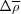 and 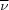 is assumed to be the same for all proteins, the mass is proportional to *I*(0) normalized by the protein concentration in (*w*/*v*) units (Mylonas & Svergun, 2007 ▸). However, the simplest implementation of this ratio method is not readily applicable to polynucleotides or protein–polynucleotide complexes. Also, for proteins experimentally determined values of 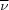 vary by as much as 10%. For a typical folded and hydrated protein, 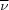 is in the range 0.70–0.74 cm^3^ g^−1^ (Harpaz *et al.*, 1994 ▸), and hydration, flexibility or modifications such as glycosylation can affect the value. The value of 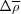 also can vary, especially in the case of bound metal ligands, for example. Additionally, it is the case that most readily available inexpensive protein standards have some tendency for time-induced and/or radiation-induced aggregation or degradation, which introduces further systematic error in the assessed *M* value. Nevertheless, it can be useful in practice to measure a known protein standard (such as lysozyme, bovine serum albumin or glucose isomerase) as a check of the overall experimental setup. However, we do not recommend dependence on this approach for the evaluation of *M* in favour of absolute scaling of the SAS data and using (1), as this method is subject to fewer errors.

The total scattered intensity [calculated as the integral from zero to infinity of *q* ^2^ *I*(*q*)] is referred to as the Porod invariant *Q_i_*, which, for uniform scattering density particles with a well defined boundary, depends only on the volume of the scattering particle and not its form (Porod, 1951 ▸). The particle volume or Porod volume, *V*_P_, is then calculated as
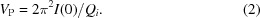
As *Q_i_* is an integral from zero to infinity and data are only measured for a finite *q* range, in practice the integral is generally estimated using a smoothed, regularized scattering profile obtained from *P*(*r*) [for example as in the method of Fischer *et al.* (2010 ▸) and in the current implementation of *GNOM* (Petoukhov *et al.*, 2012 ▸)]. The *GNOM* implementation includes a correction to force the high-*q* data to obey the expected *q* ^−4^ dependence for a uniform scattering density particle with a well defined boundary (*i.e.* a globular, folded biomolecule; Porod, 1951 ▸). By interrogating a large set of theoretical scattering profiles calculated from coordinates of proteins in the Protein Data Bank (PDB; Berman *et al.*, 2000 ▸), Fischer and coworkers determined empirical correction factors for estimating *Q_i_* for scattering data acquired over specific measured *q* ranges. Rambo and Tainer defined a new invariant that does not depend upon the *q* ^−4^ assumption and thus is applicable to both folded, globular molecules and flexible systems, the latter of which have a shallower *q* ^−3^ or *q* ^−2^ dependence (Rambo & Tainer, 2013*b* ▸). This invariant can be used to calculate a volume of correlation, *V*_c_. Any one or all of these methods can be used to estimate the volume of the scattering particle, which can then be related to *M*, keeping in mind that they all are highly dependent on accurate background subtraction. A useful rule of thumb for the ratio *V*_P_/*M* is ∼1.45–1.50. Agreement of this estimate with that derived using (1) and with the expected value from the chemical composition of the particle of interest (full sequences, including tags, bound ligands and modifications) is a primary validation parameter that demonstrates that the scattering particle is a monodisperse, folded macromolecule or macromolecular complex, and that the SAS data are suitable for quantitative structural interpretation and three-dimensional modelling.

In the case of SANS with contrast-variation data, *I*(0) and *R*_g_ values vary with contrast and hence should be reported for each contrast point measured. The *M* or *V* estimate from *I*(0) should also be determined for each contrast point to identify potential ^2^H_2_O-induced aggregation effects [from (1), for a constant *M* and 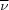, *I*(0) ∝ 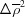]. In addition, the Stuhrmann plot (*R_g_* ^2^ *versus* 1/Δρ; Koch & Stuhrmann, 1979 ▸) is valuable to show as it provides information on internal scattering density variations within the scattering particle. For a particle composed of discrete components with distinct mean scattering densities (for example a protein plus polynucleotide, or ^2^H-labelled and unlabelled proteins) a combination of the Stuhrmann analysis and application of the parallel axis theorem (Engelman & Moore, 1975 ▸) will provide information on the disposition of components, the *R*_g_ values of each component and the *R*_g_ value for the total particle at infinite contrast (*i.e.* where internal scattering density fluctuations are negligible; Whitten *et al.*, 2008 ▸). With sufficient measurements in the contrast series it is possible to extract the scattering profiles for individual components along with a cross-term that encodes information on the dispositions of the components. The *MULCh* suite of programs (***M**od**UL**es for the analysis of **C**ontrast variation data*; available for download and as a web-based tool at http://smb-research.smb.usyd.edu.au/NCVWeb/index.jsp; Whitten *et al.*, 2008 ▸) was designed to aid in planning a SANS contrast-variation experiment by providing the dependence of *I*(0) on contrast for given deuteration levels in biomolecular components and solvent (Contrast module), for Stuhrmann and parallel axis theorem analysis (Rg module), and for extraction of the scattering profiles of individual components of a complex and their cross-term (Compost module).

The above *q* ^−4^ approximation for the decay of high-*q* data is a reasonable approximation for most folded proteins, but not for unfolded proteins, where for a fully random-coil chain the dependence is *q* ^−2^ (Debye, 1947 ▸). The asymptotic behaviour of the high-*q* data thus can distinguish between folded, partly flexible and unfolded structures. Where flexibility is a possibility, its qualitative evaluation can be made using Kratky [*q* ^2^ *I*(*q*) *versus q*; see chapter 11 of Glatter & Kratky, 1982 ▸] and Porod–Debye [*q* ^4^ *I*(*q*) *versus q* ^4^; Debye *et al.*, 1957 ▸] plots of the data (recently reviewed in Rambo & Tainer, 2011 ▸), provided that background subtractions are accurate. The dimensionless Kratky plot [(*qR*_g_)^2^ *I*(*q*)/*I*(0) *versus* *qR*_g_] is most useful to distinguish between different degrees of folding. Proteins containing folded domains display a bell-shaped curve, with a maximum of about 1.1 at around *qR*_g_ = 1.75. With increasing elongation and degree of unfolding, the maximum shifts to the upper right and the upward slope of the right side of the curve increases (Durand *et al.*, 2010 ▸; Bizien *et al.*, 2016 ▸).

Presentation of the data, analysis and validation parameters as recommended in the summary in Table 3 ▸ will aid both the experimenter and the reader in evaluating data quality, the validity of the analysis and the suitability of the data for structural modelling. The recommendations include depositing the data in a publically available archive.

### 2.4. Structure modelling   

Having obtained accurate and sufficiently precise data as *I*(*q*) *versus q* for the system of interest, provided evidence that the scattering profile is free of nonspecific aggregation or interparticle interference effects, that it yields the expected *M* or *V* value, and having assessed the potential flexibility of the system, a three-dimensional modelling strategy can be selected. This strategy may include *ab initio* shape or bead modelling and/or atomistic modelling using domains or subunits of known structure, usually derived from crystallo­graphy or NMR experiments and potentially additional experimental restraints. The model is optimized such that a penalty function is minimized that includes the fit to the scattering data (*i.e.* χ^2^) and any other penalties related to restraints on the model (*e.g.* compactness, connectedness, distance restraints *etc.*).

As solution scattering data reduce to one-dimensional profiles, there are a number of issues regarding the representation and precision of derived three-dimensional models (Schneidman-Duhovny *et al.*, 2012 ▸). In the case of data that can be adequately fitted by a single average three-dimensional model (either shape or atomistic representations), an evaluation of the inherent ambiguity in the modelling solution is required. Here, a question to answer is whether a single best-fit model or class of very similar models uniquely fits the data, or whether multiple classes of models exist that fit the data equally well. *AMBIMETER* is a recently released program that provides an *a priori* assessment as to whether the spherically averaged single-particle scattering can be fitted by a single relatively well-defined shape, or whether it is consistent with multiple shapes (Petoukhov & Svergun, 2015 ▸). It is common practice to run multiple independent model optimizations with SAS data and to use a cluster analysis to compare models in terms of their shape or, in the case of atomistic models, relative positions and orientations of domains or subunits and contacts between the different components. Providing that conformational space has been adequately sampled, the number of clusters that fit the data provides an estimate of the ambiguity in the model solution. Spatial restraints from complementary experiments (for example symmetry, domain structures from NMR or crystallography, distances or orientational restraints from chemical cross-linking, NMR, Förster resonance energy transfer, sequence conservation or co-variation) can be imposed as part of any modelling strategy to increase the resolution of the model representation and its precision (Schneidman-Duhovny *et al.*, 2012 ▸; Rambo & Tainer, 2013*a* ▸). An outstanding question in ongoing research with regard to hybrid atomistic modelling is whether the conformational search space is adequately sampled and how this can be achieved.

Symmetry assumptions in bead or shape modelling can highly influence the resulting models, and thus if symmetry is imposed to generate a model that is to be used, it is advisable to compare the result obtained in the absence of symmetry restraints. In the event that the imposition of symmetry results in a shape that is radically different to shapes derived without the symmetry assumption, the symmetry assumption may be incorrect.

If monodispersity in solution cannot be achieved or guaranteed, the measured scattering intensity reflects the spherical average over all *K* species present. Assuming non-interacting particles, the scattering intensity is then a linear combination of the scattering of the species *I_k_*(*q*) multiplied by their respective number density *n_k_*, 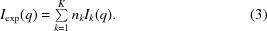
Depending on the number of components in the solution, there are various approaches to data analysis. In the case of mixtures with a limited number of components whose individual scattering intensities are known, the population fractions may be estimated from (3) (for example using the program *OLIGOMER*; Konarev *et al.*, 2003 ▸). For systems with unknown structure existing in a stable equilibrium, for example a monomer and dimer with known association and disassociation constants, three-dimensional structural analysis is possible. This can be performed *ab initio* or using rigid-body modelling (for example with *GASBORMX* or *SASREFMX*; Petoukhov *et al.*, 2013 ▸). The reporting guidelines for using these programs are similar to the monodisperse case but with the extra parameter of the fraction of each species in solution, and typically multiple curves are recorded for analysis (*e.g.* a concentration series).

Perhaps the most complicated mixtures are flexible systems containing multiple conformers, for example multidomain proteins with flexible linkers or hinges. For such systems, the number of terms in (3) can be astronomically high. These systems may still be characterized with multistate or ensemble methods where a large population of potential conformations is generated and substates or sub-ensembles that describe the observed scattering data based on *a priori* information are selected (Tria *et al.*, 2015 ▸; Berlin *et al.*, 2013 ▸; Schneidman-Duhovny *et al.*, 2016 ▸; Perkins *et al.*, 2016 ▸; Kikhney & Svergun, 2015 ▸; Terakawa *et al.*, 2014 ▸; Pelikan *et al.*, 2009 ▸; Yang *et al.*, 2010 ▸; Bernadó *et al.*, 2007 ▸). As the number of degrees of freedom in ensemble modelling is so much larger than when optimizing a single average model, the danger of overfitting and over-interpretation is significantly amplified. Satisfactory solution of the problem of multistate/ensemble modelling thus depends greatly on the application of restraints from complementary experiments or bioinformatics to limit the conformational space that must be sampled. While many programs for multistate/ensemble modelling produce representative structures to describe the range of states within the population, these representative structures are generally neither accurate nor precise in their detail and primarily aid in providing a visual, qualitative description of the nature of representative states. On the other hand, the distribution of *R*_g_ values for the optimized ensemble is generally quite robust, providing a quantitative measure of the extent of structural flexibility (Bernadó *et al.*, 2008 ▸; Carter *et al.*, 2015 ▸). In cases where the conformational space is sufficiently restrained and exhaustively sampled, it may be practical to evaluate the ambiguity and precision of the multistate/ensemble models. For example, consider a system where the data are explained by ‘open’ and ‘closed’ structural states. A cluster analysis on the opened and closed states may reveal little variability in the closed state, and thus low ambiguity and higher precision, while the open structure may show larger variation and consequently high ambiguity and low precision (see, for example, Fig. 3J in Carter *et al.*, 2015 ▸).

For atomistic representations, the protocol used to include contributions to the scattering data from the hydration layer is important. These effects are quite significant for SAXS and for SANS from samples with high levels of D_2_O (Kim & Gabel, 2015 ▸; Zhang *et al.*, 2012 ▸; Svergun *et al.*, 1998 ▸; Perkins, 1986 ▸). They become especially significant and important to report in the co-refinement of SAXS/NMR data for solution structure determination (Grishaev *et al.*, 2010 ▸).

The most commonly used parameter for evaluating the discrepancy between the scattering profile computed from a model and the measured scattering profile is the global fit parameter χ^2^, which is defined most simply as
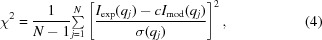
where *N* is the number of points in the scattering profile, *I*_exp_(*q*) is the experimental scattering profile, *I*_mod_(*q*) is the computed scattering profile based on the three-dimensional model, *c* is a multiplicative scaling parameter that is used to minimize χ^2^, and σ(*q*) is the standard error for each measured data point. From (4) we see that χ^2^ will be smaller for data with poor statistics and conversely larger for data with vanishingly small statistical errors. Thus, while relative χ^2^ values are most valuable in comparing two models against the same data set, absolute values can be less useful in comparing fits to two independent data sets.

Scattering data are acquired as the sum of events on a detector. A model that fits the data within its error estimates will have a χ^2^ value close to 1, providing that the random statistical errors are propagated correctly and there are no systematic errors. Overestimation or underestimation of the statistical errors and potential contributions from systematic errors have led to reported χ^2^ values ranging from a few tenths to quite large values (>5), and yet the fits to the data may be good, even excellent, or claimed to be good based on a ‘by-eye’ evaluation of a presented plot (see, for example, Supplementary Fig. 2 in Appolaire *et al.*, 2014 ▸). Generally, SAS intensity decreases rapidly and by orders of magnitude over the measured *q* range, and depending upon how the data are presented, regions of significant misfitting of the scattering profile may not be apparent. Also, as χ^2^ is a global fit parameter, it is important to present the data and model fit so that systematic deviations that may be present in specific *q* regimes are evident, for example in the mid-*q* regime most highly influenced by domain positioning and orientation where SAS data are often most helpful in SAXS/NMR structure refinement (Grishaev *et al.*, 2008 ▸). A straightforward and intuitive approach to demonstrating the quality of a model fit over the entire measured or modelled *q* range of a SAS profile that takes into account relative errors across the measured *q* range is an error-weighted residual difference plot of [*I*_exp_(*q*) − *cI*_mod_(*q*)]/σ(*q*) *versus q*, as is nicely demonstrated in Figs. 3, 4 and 5 of Carter *et al.* (2015 ▸). The error weighting of this difference plot aids in visualization by preventing the plot from being dominated by regions of weaker scattering and poor statistics. This plot presents the fit in the noisy high-*q* regions without losing information in the low- to mid-*q* regions that contain the shape information that can be most important for biomolecular SAS modelling. If the deviations from the model are only evident in the high-*q* regime, it might be indicative of an error in solvent subtraction or unaccounted-for disorder.

Different modelling programs use various adjustable parameters in their procedures to minimize χ^2^ and these are valuable to consider (*e.g.* for *CRYSOL* the parameters Vol, Dro and Ra specify the excluded volume, scattering density contrast in the hydration layer and atomic group radius, respectively, and there is also an optional adjustable constant term to account for possible errors in the solvent subtraction; for *FoXS* the parameters *c*_1_ and *c*_2_ are used to adjust excluded volume and hydration-layer density to account for the hydration layer). Understanding these parameters is necessary to ensure that they represent realistic assumptions given the physics of the system. Here, it should be noted that not only do different modelling programs use different adjustable parameters, they sometimes evolve over time in ways that can affect the absolute value of χ^2^; for example, a later version may incorporate an adjustable constant subtraction/addition for optimization which can significantly affect χ^2^.

The different detector characteristics, protocols for error propagation, details of the modelling algorithm and nature of the adjustable parameters renders comparisons of published χ^2^ values from different experiments and different modelling calculations performed at different points in time essentially meaningless. Alternative statistics have been proposed, including a Pearson correlation-based method (dos Reis *et al.*, 2011 ▸) and a measurement of the volatility of the ratio between experiment and fit (Hura *et al.*, 2013 ▸). Rambo and Tainer proposed the use of a resampling-based adaptation of the reduced χ^2^ test and defined a χ^2^_free_ with the aim of reducing the chance of model misidentification in noisy data and avoiding overfitting (Rambo & Tainer, 2013*b* ▸). The χ^2^_free_ parameter, however, does not solve problems relating to inaccurate error propagation. A recently proposed alternative to χ^2^ that is independent of the amplitude of the statistical errors considers only the statistical likelihood of a run of consecutive points lying systematically above or below the profile generated from the fitted model (Franke *et al.*, 2015 ▸). The method has proven to be useful for comparing synchrotron SAXS data frames to detect subtle radiation damage or for selecting SEC–SAXS data frames for averaging and subsequent analysis. As implemented in *ATSAS*, a two-dimensional correlation map (*CORMAP*) is generated that usefully highlights patterns of systematic deviation. A score (*P*-value) is assigned relating to the statistical probability of the longest run of points that lie consistently above or below the model. While *CORMAP* does not require knowledge of errors, if the random errors are very small and because the model curve is smooth, a constant sign of difference can easily be observed over a long *q* range, resulting in very small *P*-values. In such cases of data with high statistical precision, χ^2^ would also be expected to be greater than 1 owing to systematic deviations between the experimental data and model curve.

The above issues and limitations noted, χ^2^ nonetheless remains an accepted and necessary parameter to report as most modelling protocols minimize χ^2^ one way or another. However, reporting a combination of χ^2^ values with comments on the confidence level with which a global minimum was identified along with a clear graphical representation of deviations between the model and the experimental data in the form of a residual plot is essential.

Assessing the precision, or variability among all sufficiently well scoring models, is important for SAS-derived models. Recently, a tool has been developed that uses the Fourier shell correlation criterion widely employed in electron-microscopy model assessment to evaluate the variability among *ab initio* shape models to provide an assessment of the model precision in terms of a resolution (Tuukkanen *et al.*, 2016 ▸). The method (*SASRES*) is implemented in the bead-modelling tools of the *ATSAS* package (Petoukhov *et al.*, 2012 ▸). A clear benefit of this tool is that it will discourage the over-interpretation of surface bumps and valleys in these models.

For a given optimized atomistic model, accuracy will vary substantially for different regions depending on the contributing data. For example, the linker sequences between structured domains from crystallography or NMR that are modelled only by optimizing the fit to the SAS data will not be accurate at the level of coordinate positions. Likewise, interfaces that are not defined experimentally by crystallography or NMR are likely not to be accurate. The disposition of the domains may be relatively well defined; that is, accurate within limits that can be placed on the spatial and orientational parameters (Kim & Gabel, 2015 ▸; Gabel, 2012 ▸). The accuracy will depend on the asymmetry of the structure shape and whether there were additional contacts from experiment or bioinformatics analysis used as restraints. Their precision can be estimated from the variability of equally scored models providing that conformational space was exhaustively sampled. It is thus important in reporting atomistic models to clearly identify the sources of the components of the model; where there is high-resolution information, its accuracy and precision, the basis for building regions of unknown structure and how the conformational search space was restrained to enable adequate sampling. Table 4 ▸ summarizes the recommended reporting guidelines for structural modelling.

## 3. An example: SEC–SAXS experiments on three proteins   

**Figure 1:**
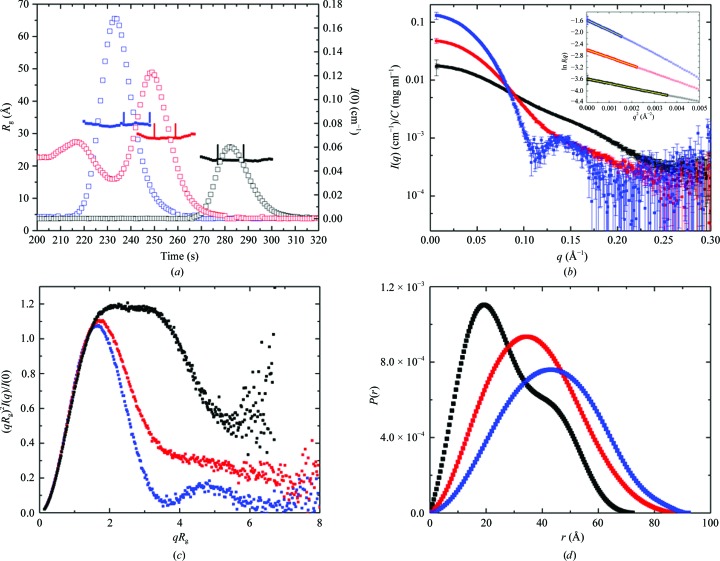
SEC–SAXS results for GI (blue), BSA (red) and CaM (black). (*a*) Plots showing *I*(0) (hollow squares) and *R*_g_ (filled squares) as a function of time for the SEC–SAXS run. Data frames between the vertical bars were selected for averaging to obtain *I*(*q*) *versus q*. (*b*) *I*(*q*) *versus* *q* as log-linear plots with the inset showing the Guinier fits (yellow lines) for *qR*_g_ < 1.3 with open symbols indicating data beyond the Guinier region. (*c*) Dimensionless Kratky plots for the data in (*b*). (*d*) *P*(*r*) *versus r* profiles from the data in (*b*) normalized to equal areas [*i.e.* proportional to *P*(*r*)/*I*(0)] for ease of comparison.

**Figure 2:**
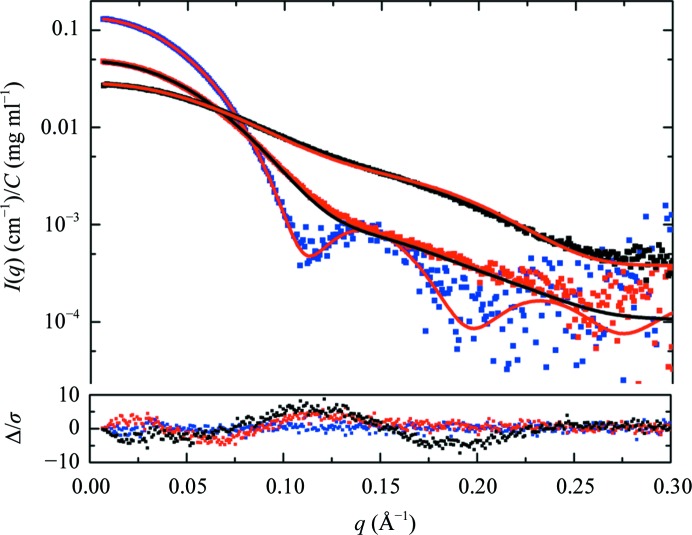
Crystal structure modelling results. *FoXS*-derived models (red and black solid lines) for GI (PDB entry 1oad, tetramer), BSA (PDB entry 4f5s, chain *A*) and CaM (PDB entry 1cll with the additional N- and C-terminal residues modelled) fitted to *I*(*q*) *versus q*. The upper plot shows log *I*(*q*) *versus q*, while the lower inset plot is the error-weighted residual difference plot Δ/σ = [*I*_exp_(*q*) − *cI*_mod_(*q*)]/σ(*q*) *versus q*. The colour key for the data plots is the same as in Fig. 1 ▸.

**Figure 3:**
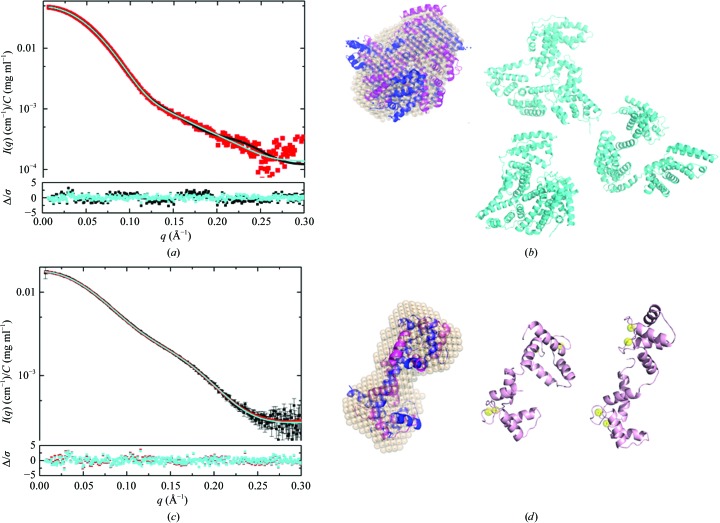
*MultiFoXS* modelling results for BSA and CaM. (*a*) Model fits for BSA: *I*(*q*) *versus q* (red squares) for one-state (black line) and three-state (cyan line) models assuming flexible residues 183–187 and 381–384. The lower inset shows the error-weighted residual difference plots for one-state (black squares) and three-state (cyan squares) models. (*b*) BSA *DAMMIN* model (wheat spheres) overlaid with the crystal structure (PDB entry 4f5s, chain *A*, blue ribbon) and one-state optimized model (magenta ribbon) and representative structures from the three-state optimized model (cyan ribbon models). (*c*) Model fits to *I*(*q*) *versus q* for CaM: *I*(*q*) *versus q* (black squares) for one-state (red line) and two-state (cyan line) models assuming flexible residues 1–3 and 77–81; the lower inset shows the error-weighted residual difference plots for the one-state (red squares) and two-state (cyan squares) models. (*d*) CaM *DAMMIN* model (wheat spheres) overlaid with the crystal structure (PDB entry 1cll, blue ribbon) and the one-state model (magenta ribbon) with the representative two-state models to the right (pink; calcium ions are depicted as yellow spheres). Model overlays were optimized using *SUPCOMB* (Kozin & Svergun, 2001 ▸).

**Figure 4:**
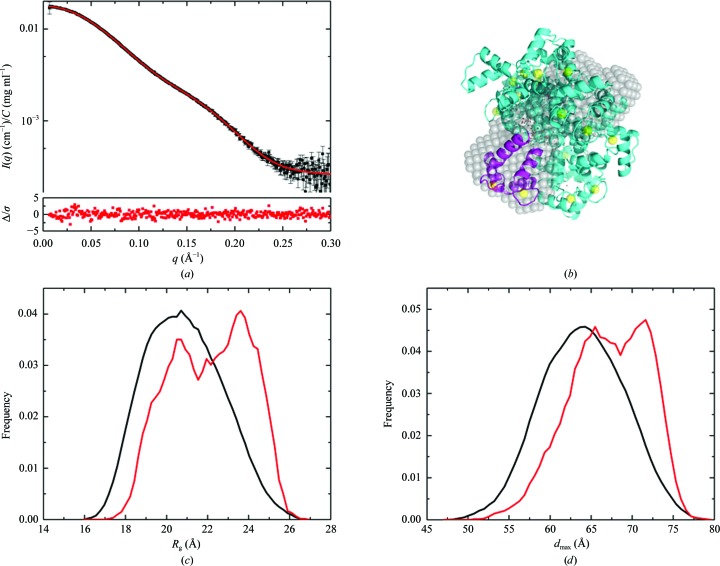
Ensemble modelling results for CaM. (*a*) *I*(*q*) *versus q* (black squares) with the *EOM* model (red line) and error-weighted difference plot for the model and experimental profiles (red squares). (*b*) Averaged and filtered *DAMMIN* model (grey spheres) overlaid with representative structures from the optimized ensemble. Structures are aligned by their N-terminal domains (magenta), showing variability in the relative disposition of the C-terminal domains (cyan). The calcium ions are depicted as yellow spheres. Given the variations in the selected structures, the overlay with the *DAMMIN* model was performed simply by eye in *PyMOL*. (*c*, *d*) *R*_g_ and *d*_max_ distributions, respectively, from *EOM* for the starting pool (black line) and the optimized ensemble (red line).

The following section, together with Figs. 1–4, Supplementary Fig. S1 and Tables 5 ▸(*a*)–5 ▸(*g*), describes the conduct and results of a set of SEC–SAXS experiments on solutions of glucose isomerase (GI; a well characterized tetramer in solution; Ramagopal *et al.*, 2003 ▸), bovine serum albumin (BSA; a two-domain protein with a flexible loop connecting its domains and known to be prone to oligomerization) and Ca^2+^-bound calmodulin (CaM; a two-domain protein known to have an extended helix with a highly mobile region linking two domains that in solution move independently; Babu *et al.*, 1988 ▸; Barbato *et al.*, 1992 ▸; Heidorn & Trewhella, 1988 ▸). The example data sets were deliberately selected to be well characterized protein structures, but not necessarily ideal measurements, in order to demonstrate how the reporting guidelines aid in both data assessment and model evaluation and in assembling a comprehensive description of the experiment and the models that the data support. The tabulated results for all three proteins provided the subset of information required for the deposition of metadata, data and models in the SASBDB (deposition IDs are provided in Table 5 ▸ *g*).

The SAXS data were acquired using the SAXS/WAXS beamline at the Australian Synchrotron (Kirby *et al.*, 2013 ▸) with a sheath-flow sample environment to maximize the X-ray dose on the sample with minimal radiation loss (Kirby *et al.*, 2016 ▸). All measured intensity values were multiplied by 2.05 to account for the shortened sample path length in the sheath-flow cell (0.49 mm) with absolute scaling calibrated to 1 mm H_2_O scattering. SAS data reduction used the beamline software *ScatterBrain* 2.82, and we note here that this version of *ScatterBrain* outputs errors that are twice the standard error and were halved before use in analysis programs. Solvent subtraction, *R*_g_, *P*(*r*) and bead modelling were performed with programs from the *ATSAS* package (Petoukhov *et al.*, 2012 ▸); *FoXS* and *MultiFoXS* were used for atomistic and multistate modelling (Schneidman-Duhovny *et al.*, 2016 ▸) as well as *EOM* for ensemble modelling (Bernadó *et al.*, 2007 ▸). The choice of different multistate/ensemble modelling approaches was simply to demonstrate the different reporting involved.

The path length between UV absorption and SAXS measurements was minimized, enabling the use of *A*_280_ measurements to estimate protein concentration for the SAXS data in the measurement frames used for analysis. Accounting for the 0.31 cm path length of the UV cell used for measurement, the *A*_280_ values are all multiplied by 3.22 for concentration determination using extinction coefficients calculated for a 1 cm path length. The *A*_280_ measurements associated with the selected SAS measurement frames (Supplementary Fig. S1*a*) for analysis were used with calculated extinction coefficients (using *ProtParam*; Gasteiger *et al.*, 2005 ▸) to estimate protein concentrations.

Guinier analysis during data acquisition (autogenerated by *PRIMUS*; Petoukhov *et al.*, 2012 ▸) yielded values of *R*_g_ and *I*(0) for each 1 s measured data frame. The *R*_g_ and *I*(0) traces (Fig. 1 ▸ *a*) as a function of time show that the GI and CaM samples are highly pure, as expected from their sources. GI was originally sourced from Hampton Research, stored in diluted form for some period and subject to repeated freeze–thaw cycles. CaM was prepared by bacterial expression and high-resolution SEC (Michie *et al.*, 2016 ▸). The commercially purified BSA powder had aged in the refrigerator for some years and the SEC trace indicated that it was highly heterogeneous, which is consistent with the known tendency of this protein to self-associate and the lack of any steps to remove higher order oligomers prior to loading.

Data frames under each of the main elution peaks for which the *R*_g_ values were the same within error and statistically indistinguishable as assessed using *CORMAP* (Franke *et al.*, 2015 ▸) were selected and averaged for further analysis. For CaM, 12 × 1 s frames centred on the maximum in *I*(0) where the *R*_g_ plot was flat were chosen. For GI, *R*_g_ showed a small increase after the peak (by an average of 0.6 Å over 9 × 1 s measurement frames) starting where the concentration dropped to ∼1 mg ml^−1^ (compared with 1.27 mg ml^−1^ in the peak). In addition, the *P*(*r*) transform that included data from the frames corresponding to the smaller *R*_g_ values showed a significant negative dip around *d*_max_ consistent with there being a weak structure-factor contribution. GI has a net negative charge at pH 7.5 and, as we have previously observed, there is a small but measurable inter-particle interference contribution to the scattering for concentrations of >1 mg ml^−1^. By selecting 11 × 1 s frames to the right of the peak, the *P*(*r*) transform showed a much reduced negative dip around *d*_max_. It is noteworthy that both CaM and GI are expected to have a net negative charge at pH 7.5, but only GI showed evidence in the scattering for inter-particle correlations owing to charge repulsion. For BSA, 10 × 1 s frames were chosen for analysis starting from the maximum recorded *I*(0) where the *R*_g_ had plateaued.

A total of 50 × 1 s frames taken prior to each protein peak were averaged for the solvent blank, although in the case of BSA this choice resulted in a slight upturn in the Guinier plot for the lowest five data points (*q* < 0.01 Å^−1^), which could arise either from a slight error in the solvent subtraction or from aggregation. Exploration of the measurements of solvent before and after the BSA elution peak indicated variation in the solvent scattering and, for BSA only, the solvent blank was taken from 50 frames after the protein had eluted. With this solvent measurement, the Guinier plot was linear to the lowest measured *q* value.

The log *I*(*q*) *versus q* plot (Fig. 1 ▸ *b*) represents the primary SAS data, with Guinier plots shown as insets. The maximum dimensions for all the three proteins are <100 Å, and the minimum *q* measured (0.007 Å^−1^) is well below the minimum of *q* ≃ π/*d*_max_ = 0.03 Å^−1^ recommended for accurate assessment of the largest particle (GI). Importantly, for all three proteins the Guinier plots are linear to the first measured *q* values (Pearson *R* values of 0.999) and a plot of log *I*(*q*) *versus* log *q* (Supplementary Fig. S1*b*) shows that the slope is effectively zero at low *q* as expected for monodisperse particles of similar size. These measures together provide confidence that the data are free of significant amounts of contaminating species or inter-particle correlations contributing a structure-factor term to the scattering.

Dimensionless Kratky plots (Fig. 1 ▸ *c*) demonstrate that the SAS data are from predominantly folded particles. The GI and BSA plots display the expected bell-shaped curve, with a maximum of about 1.1 at around *qR*_g_ = 1.75. The peak for BSA is slightly shifted to the right as expected for its slightly elongated shape, and the small rise evident at *qR*_g_ > 7 suggests some flexibility. The more elongated dumbbell-shaped CaM gives rise to a distinct profile. The maximum on the vertical axis for CaM is somewhat higher than the expected 1.1 and is shifted to *qR*_g_ = 2 because of its elongated shape, while the shallow oscillation at 2.5 < *qR*_g_ < 3.5 reflects the well resolved two-domain structure. As expected for CaM, significant flexibility is indicated by the increase in intensity at *qR*_g_ values of >6. For comparison, Supplementary Fig. S1(*c*) shows the standard Kratky plot, from which similar conclusions can be drawn regarding flexibility.

The *P*(*r*) *versus r* profiles for each of the proteins (Fig. 1 ▸ *d*) are well behaved, showing the smooth, concave approach to zero at *r* = 0 and *d*_max_ expected for a mostly folded, monodisperse protein. The *P*(*r*) profiles also have the expected characteristics based on the available crystal structures: a single major peak for the globular GI and BSA structures and the peak and shoulder expected for the dumbbell-shaped CaM.

For all three proteins, the *R*_g_ and *I*(0)-based *M* values [using (1)] are in excellent agreement between independent Guinier and *P*(*r*) analyses (Table 5 ▸ *d*). For the GI tetramer and BSA, the *M* values estimated from *I*(0) are all within 1–4% of the expected values based on chemical composition. On the other hand, the *M* values for CaM are ∼30% larger than that expected for the monomer, which is large even considering that calculated extinction coefficients for non-Trp-containing proteins can be >10% (Gasteiger *et al.*, 2005 ▸). However, the ratio *V*_P_/*M* calculated from the chemical composition for BSA and CaM is 1.5, and is slightly on the small side for GI at 1.3, perhaps indicating that there was still some residual inter-particle interference in these data, for which there was also a small residual negative dip in the *P*(*r*) transform around *d*_max_. The *M* values determined using the Fischer–Porod method (Fischer *et al.*, 2010 ▸) in kDa with their ratios to the expected value in parentheses were 157.9 (0.91), 67.9 (1.02) and 17.7 (1.05) for GI, BSA and CaM, respectively. The Porod-derived *M* value for GI is again low, while those for BSA and CaM are within 2–5% of those expected. For CaM, it thus appears that potential errors in the concentration owing to its relatively weak extinction coefficient and/or in 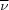 and 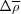 based on chemical composition for this relatively small (<20 kDa) and flexible protein results in an overestimation of *M* from *I*(0).

The *R*_g_ values for GI and CaM (Table 5 ▸ *d*) are in good agreement with previously published values from SAXS measurements [Guinier *R*_g_ values of 32.5 ± 0.7 Å for GI (Mylonas & Svergun, 2007 ▸) and 21.0 ± 0.6 Å for CaM (Heidorn & Trewhella, 1988 ▸)], whereas the value for BSA lies in between a previously published value from SAXS (29.9 ± 0.8 Å; Mylonas & Svergun, 2007 ▸) and that predicted from the crystal structure (26.75–26.89 Å using *FoXS* or *CRYSOL*) from the individual monomer chain *A* in the dimeric crystal structure (Table 5 ▸ *f*).

For all three proteins, the *ab initio* bead-modelling program *DAMMIN* (Svergun, 1999 ▸) was better able to fit the data than its speedier cousin *DAMMIF* (Table 5 ▸ *e*). However, the latter program provides a rapid assessment of the variability of the shapes that fit the data from 20 independent calculations using the normalized spatial discrepancy (NSD) value. The NSD value is ≤0.7 for GI, indicating largely similar shapes, but is >0.7 for BSA and CaM, which is suggestive of distinct classes of shape, and a cluster analysis identified four and six sub­classes for BSA and CaM, respectively. The relatively high χ^2^ values for the *DAMMIF* models for GI are largely owing to misfitting around the local minimum in this profile just above *q* = 0.1 Å^−1^, and it is noteworthy that the *M* estimation from the *DAMMIN* calculation for GI is low, again similar to what we observe for the ratio *V*_P_/*M*. We note that the CaM data have the largest constant adjustment to intensity (by an order of magnitude compared with GI) applied to minimize χ^2^ in the uniform density bead modelling, likely owing to the known flexibility in CaM. The adjustment for BSA is intermediate.

As there are crystal structures for all three proteins, atomistic modelling was undertaken (Table 5 ▸ *f*). A tetramer based on the crystal structure of GI (PDB entry 1oad; Ramagopal *et al.*, 2003 ▸) predicts an *I*(*q*) profile that is a reasonable fit to the scattering data (see Fig. 2 ▸; χ^2^ = 1.02 from *FoXS* or 1.03–1.00 from *CRYSOL* depending on whether a constant subtraction is allowed). However, it is noteworthy here that the GI data have the poorest statistics of our three examples owing to a significant portion of the scattering being taken at lower concentrations. Given the indications of inter-particle interference that were observed, at this point the experimenter should be questioning whether the data are of sufficient reliability and statistical quality for their purposes. It is reasonable to conclude from the data that GI is a tetramer with a shape and structure that is largely consistent with the crystal structure. To go beyond making this assessment, repeating the experiment to obtain data with better statistical precision that are clearly devoid of inter-particle interference is called for.

In contrast to GI, the crystal structures of BSA (PDB entry 4f5s chain *A*) and of CaM (PDB entry 1cll) showed very poor fits to their respective data sets (χ^2^ = 4.4 and 10.8, respectively, from *FoXS*). There are a few missing amino acids in the CaM crystal structure (Ala-Asp-Gln at the N-terminus and a Lys at the C-terminus. These were added to the crystal structure (1cll+) using *MODELLER* (https://salilab.org/modeller/; Webb & Sali, 2014 ▸), and the *FoXS* χ^2^ value decreased marginally to 9.2. Interestingly, in trying to fit the CaM data to the unmodified crystal structure, the *FoXS* calculation takes *c*_2_ to its limit of 4, which corresponds to the highest permitted hydration-layer scattering density for the program (∼0.388 e Å^−3^). With the modified crystal structure 1cll+ *c*_2_ is somewhat smaller (2.94). Values that are smaller again are obtained when fitting the crystal structures of BSA (2.39) and GI (0.81). The values of these adjustable parameters can provide a warning that the calculation is trying to adjust the hydration-layer parameters for something that is likely to be missing in the model, which in the case of CaM, and possibly also BSA, we expect to be flexibility. Results for the crystal structure comparisons to the data obtained using *CRYSOL* (Svergun *et al.*, 1995 ▸) also show considerable variability in the adjustable parameters, and the χ^2^ values from *CRYSOL* are much larger for CaM, presumably because *CRYSOL* models an explicit scattering contrast from the hydration layer and the values are constrained to a particular range. The effect of the constant adjustment to intensities in the optimization that is an option in *CRYSOL* is also demonstrated; with the extra degree of freedom, smaller χ^2^ values are obtained.

The overall misfits to the crystal structures for CaM and BSA are much clearer in the error-weighted residual difference plots than in the log *I*(*q*) *versus q* plots of the model overlaid with the experimental data (Fig. 2 ▸). Both BSA and CaM are multidomain structures, and the ‘wave’ observed in the difference plot is suggestive of a shift, on average, in the relative positions and/or orientations of domains in solution compared with the crystal form.

The crystal structure of BSA shows two domains stabilized by a tight network of disulfides linked by a long flexible loop with high temperature factors assigned to residues 183–187 and 381–384 that are proposed to be responsible for domain movements (Bujacz, 2012 ▸). Multistate modelling using *MultiFoXS* and allowing for flexibility in these residues yielded a much-reduced χ^2^ of 1.05 for a one-state model and the minimum χ^2^ of 0.82 for a three-state model. The model *I*(*q*) profiles for the one- and three-state models (Fig. 3 ▸ *a*) fit within the noise, and the residual difference plots between experimental and model *I*(*q*) are significantly flatter compared with the crystal structure fit, with a clear narrowing of the difference plot for the three-state model on the vertical scale (cyan symbols against black). Representative models from the best-fit one- and three-state models are shown in Fig. 3 ▸(*b*), with the bead model from *DAMMIN* overlaid with the one-state model and the crystal structure. From the weighting parameters, we see that the optimization has yielded the lowest weights to the more extended structures. Thus, the multistate modelling is supportive of the conclusions drawn from the temperature factors in the crystal structure. However, if one were looking to independently prove the presence of flexible regions, the variability in solvent scattering before and after elution of the BSA sample presents a degree of uncertainty. This uncertainty should be removed by repeating the measurements starting with freshly purchased or purified BSA that was subjected to SEC immediately prior to SEC–SAXS.

Accounting for the missing N- and C-terminal residues and the known flexibility in the extended helix that connects the two globular domains of CaM [from NMR relaxation (Barbato *et al.*, 1992 ▸) and solution SAXS (Heidorn & Trewhella, 1988 ▸)], *MultiFoXS* yields a χ^2^ value of 0.85 with a one-state model in which the CaM domains are on average reoriented compared with the crystal structure to yield a slightly more compact average *R*_g_ of 21.03 Å, and a further decrease in χ^2^ to 0.79 is obtained with the two-state model that includes structures with *R*_g_ values of 22.32 and 19.47 Å representing ∼70 and ∼30%, respectively, of the population. The error-weighted residual plots for these fits are quite flat, with a barely distinguishable narrowing of the residuals for the two-state model (Figs. 3 ▸ *c* and 3 ▸ *d*). There was no improvement in χ^2^ for the three-state model. The alternate ensemble modelling program for flexible systems (*EOM*; Bernadó *et al.*, 2007 ▸) was also used to model CaM with the same flexible residues, yielding a χ^2^ value of 0.82 (the model fit is shown in Fig. 4 ▸ *a*). As for the multistate fits from *FoXS*, the residual difference plot between experimental and model *I*(*q*) is flat, but 13 representative structures were selected to represent the ensemble (Fig. 4 ▸ *b*) and this greater structural diversity in the model is reflected in very broad distributions for *R*_g_ and *d*_max_ (Figs. 4 ▸ *c* and 4 ▸ *d*, respectively) in the optimized ensemble.

The atomistic modelling thus supports the conclusions from the dimensionless Kratky plots that BSA and CaM are both mostly folded proteins with some flexibility, which is significantly greater for CaM, and in each case assuming the flexible regions identified by crystallography or NMR improved the model fits to the data. Of note, the *P*-values obtained from the *CORMAP* analysis (Franke *et al.*, 2015 ▸) support the ranking of goodness of fit for the modelling based on χ^2^. Interestingly, the χ^2^ values for the best-fit models all fell within a relatively narrow range (0.79–1.05). In contrast, the *P*-values varied by an order of magnitude even though the accompanying changes in the length of contiguous points lying on one side of the model fit are relatively small compared with the number of points in the data set (for CaM it was ten points at ∼0.165 Å^−1^ *versus* eight points at ∼0.03 Å^−1^ for the one-state *versus* two-state models, respectively; for BSA it was 14 points at ∼0.2 Å^−1^, 12 points at ∼0.01 Å^−1^ and 11 points at ∼0.25 Å^−1^, respectively). For BSA, the differences appear to be quite subtle, and further they occur in the lowest *q* and high-*q* regimes, unlike the statistically superior CaM example where for the one-state model at least, the locus is in the mid-*q* regime that we expect to be most sensitive to domain dispositions.

## 4. Conclusions   

The example SEC–SAXS experiments on GI, BSA and CaM illustrate the value of comprehensive reporting so that data quality and model accuracy are clearly communicated. Supplementary Table S1 provides a guide for tabulating the recommended information for a general SAXS experiment; such a table will be included in future releases of the IUCr Journals Word template. Some publishers may well require much of the reporting to be included as supplementary material. Eventually, most of it should be made available *via* the developing SAXS data and model archives. The latter will be increasingly important for managing related data sets, although Figs. 2, 3, 4 and 5 in Carter *et al.* (2015 ▸) show how effectively one can assemble the results for multiple data sets.

It is evident that the often-ignored adjustable parameters enhance the understanding of potential limitations in models. In this regard, it is noted that for some programs it is not straightforward to relate the adjustable parameters to the physical model. It would be desirable for the developers of programs for SAS modelling to make information on the adjustable parameters more transparent and their values readily available in standard output formats.

The three data sets analyzed highlight advances in SEC–SAXS and the analysis of multistate ensembles. Both the GI and BSA samples were not subjected to purification steps before loading onto the SEC–SAXS column. For GI the data statistics were relatively poor, and there was evidence of incompletely removed inter-particle interference in the scattering. For BSA there were issues with the solvent subtraction. These limitations were transparent in the reporting and the modelling and interpretation appropriate in that context. For experiments aimed at hybrid modelling, for example improving the solution structure by co-refinement with NMR data, these limitations would be unacceptable and the SAS experiments should be repeated after taking steps to purify the proteins before SEC–SAXS and to optimize the conditions to obtain better quality data that are free of the issues encountered.

The CaM sample was highly purified and well characterized before SEC–SAXS and as a result delivered a superior data set in spite of its relatively small size and hence weaker total scattering power. CaM is a well characterized protein structurally, including its regions of flexibility, and the SAXS data were well fitted using multistate/ensemble modelling. An open question for multistate/ensemble modelling is whether to present the minimum number of structures that the data can support, or whether one should assume that flexible sequences will sample a continuous distribution of conformations and so a larger number in the representative set may be justified. At this time, a variety of programs allow investigators to choose their preferred multistate/ensemble modelling approach and assumptions.

Accurate propagation of uncertainties is an important area for further work in the community for SAS data to contribute to integrative/hybrid modelling. For synchrotron SAXS data, the increasing brightness of the sources has reduced the relative random statistical errors in the data to the extent that they may no longer dominate and systematic errors can become significant. A recent model has been proposed and tested for optimizing experimental setups and taking into account not just random statistical errors, but those originating from setup geometry and the physics of the measurement process (Sedlak *et al.*, 2017 ▸). The χ^2^ values near 1 for the best-fit models in our example set were all near the expected value for a fit within the random statistical errors propagated, and notably the superior CaM sample with its statistically superior data set resulted in models with the lowest χ^2^ values and no evidence of systematic errors owing to sample issues or solvent mismatch.

The error-independent *CORMAP* *P*-value for model fits correlated well with the χ^2^ values, showing a much larger range of variation. Broader experience with a large number of examples is needed to provide a basis for understanding the significance of the absolute value of the *P*-values in the context of SAS modelling. We therefore encourage experimenters to use the *CORMAP* analysis and to report the *P*-values. Once a sufficiently large sample size has been acquired, a systematic review and evaluation of their utility in the context of SAS modelling will be possible.

As biomolecular SAS continues to grow in popularity and further develop in this era of integrative/hybrid methods for the structure determination of increasingly complex bio­molecular complexes and assemblies, it is essential to firmly establish publication guidelines with the goal of ensuring access to the information required for proper evaluation of the quality of SAS samples and data, as well as the validity of structural interpretation. In addition to our recommended guidelines for data presentation in a publication, we recommend that SAS data and models be deposited and made freely available in a public data bank [currently there is SASBDB and BIOISIS (http://www.bioisis.net/)]. Ideally *q*, *I*(*q*) with standard errors should be deposited for each measured profile and the associated models plus details of how the experiment was conducted with the data and model validation parameters and analyses as outlined above. We strongly recommend that the sasCIF dictionary be expanded to include all of these data items in the recommended guidelines and encourage program developers to use the sasCIF as an export format which will significantly ease the burden on researchers in reporting, and will facilitate more automated deposition SAS databases that can support integrative/hybrid models (Sali *et al.*, 2015 ▸). Utilizing the sasCIF will also enable seamless data exchange and interoperability with other structural biology data resources, including the Protein Data Bank.

## Supplementary Material

Reporting Template for tabulating essential SAS data acquisition, sample details, data analysis, modelling fitting and software used plus a figure showing alternate data-presentation plot.. DOI: 10.1107/S2059798317011597/jc5010sup1.docx

## Figures and Tables

**Table 1:** Summary of guidelines for sample details

Source of samples, including sample-purification protocol, a measure of the final purity and how it was determined.
Composition of the sample, including protein or nucleic acid sequences as measured, or FASTA IDs with the relevant ranges specified, plus fusion tags, ligands, cofactors, glycosylation or other modifications and the predicted molecular mass.
Solvent/buffer pH and composition, including additives such as free-radical scavengers used to minimize the effects of radiation damage during SAXS data acquisition, and a statement of how the SAS-measured solvent blank was obtained (*e.g.* last-step dialysate, concentrator or column flowthrough).
Sample concentration(s) and method(s) of determination, including extinction coefficients and wavelengths when UV absorbance measurements are used.
In the case of combined SEC–SAS experiments, a description (or reference) to the system, column size/type/resin, injection sample concentration and volume and flow rate.
In the case of SANS contrast-variation experiments, the deuteration level of each biomolecular component (*e.g.* from mass spectrometry) and of the solvent (*e.g.* from densitometry or transmissions).
Any SAS-independent assessments of monodispersity over a range of conditions (*e.g.* analytical ultracentrifugation, dynamic light scattering and/or aggregate-free gel filtration and/or multi-angle laser light scattering) that complement the SAS-based assessments.

**Table 2:** Summary of guidelines for data acquisition and reduction

Instrument type (*e.g.* manufacturer and model designation or beamline) specifying the source (sealed tube, rotating anode, metal jet, synchrotron, spallation neutron source or reactor) and the configuration used (point or line source, collimation details, detector details). In the case of SANS there may be several configurations (*e.g.* multiple detector positions, number of guides, apertures *etc.*) for a single experiment.
Beam dimensions and wavelength resolution (Δλ/λ) with data-smearing parameters where appropriate, and measured *q* range including *q*_min_ limit owing to instrument resolution and beam-stop size.
References to documentation for detector type and characteristics including pixel size, the basis for error estimates and propagation (*e.g.* Poisson counting statistics) and the confidence interval represented by the errors, methods for detector sensitivity and linearity corrections.
Number of sample exposures and exposure times, the normalization method (*e.g.* time or beam monitor counts), the method used to determine sample transmission and how radiation damage was monitored (in the case of SAXS).
In the case of SANS contrast-variation experiments, sample and buffer transmissions referenced to transmissions of pure ^1^H_2_O and ^2^H_2_O, from which deuteration of the solvent can be checked.
Details of the sample environment, including measurement temperature, measurement cell type and path lengths, any special parameters controlled, *e.g.* pressure, and additional inline purification or characterization capabilities as appropriate.
In the case of SEC–SAS experiments, description of (or reference to) system.
Standards measured and controls and method for placing SAS data on an absolute scale in cm^−1^, *e.g.* by reference to a well characterized standard such as H_2_O or glassy carbon or the incident beam flux. As appropriate, any standard protein measurement used as an overall check of the experimental setup.
Data-reduction protocol and software used, including version number.

**Table 3:** Summary of guidelines for data presentation, analysis and validation

Difference scattering profiles [(particle + solvent) − (solvent scattering)] corresponding to the particle form factor deposited in a publicly available archive or made available as supplementary material and presented as a plot of log *I*(*q*) *versus q* or log *I*(*q*) *versus* log *q* along with a Guinier plot with the following.
(i) Intensities on an absolute scale in units of cm^−1^ with propagated standard errors (σ). Note: for Guinier plots [ln *I*(*q*) *versus q* ^2^] a first-order approximation to the error in ln *I*(*q*) is σ*I*(*q*)/*I*(*q*).
(ii) For multiple curves on the same plot, data can be offset for clarity with the offsets given in the figure caption.
(iii) For SANS contrast-variation experiments, data from all contrast points.
(iv) Guinier *R*_g_ and *I*(0) values with errors, a quality-of-fit parameter (such as a coefficient of correlation *R* ^2^) with the *q* or *qR*_g_ range specified and linear fits displayed with *q*_min_ < *q* ≃ π/*d*_max_. Any data from the measurement range that was truncated should be displayed and identified by the use of a symbols that distinguish them from data points included in the linear fit.
*P*(*r*) *versus r* with associated *R*_g_ and *I*(0) (with errors) and *d*_max_ values is essential for SAXS data and is advised for SANS data [especially at solvent match points for complexes of components with distinct scattering densities where interpretation of *P*(*r*) will be the most intuitive as the scattering object has an approximately uniform scattering density].
*M* or *V* estimates, preferably from multiple methods; for example, methods based on *I*(0) in addition to *V*_P_ or *V*_c_. For *I*(0)-based methods, values and uncertainties in the calculated or experimentally determined concentration and parameters used, such as 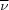 , 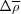 and solvent and particle scattering-length densities, along with the methods of calculation or measurement.
Where applied, the magnitude of corrections for solvent subtraction applied to the data as a potential warning that something is not correct if unduly large (say 1% percent of the solvent scattering level).
Where relevant, the method of data desmearing to correct for beam geometry and/or polychromaticity and the original smeared data be made available.
For a concentration series, note if no change in *R*_g_ or *I*(0)/*C* is observed with increasing concentration [*C* in (*w*/*v*)] and for best practice report *M* estimates at each concentrations or provide a plot of *I*(0)/*C versus C*.
A dimensionless Kratky plot as a check on the degree of folding and/or flexibility in the scattering particle. Kratky and/or Porod–Debye plots might alternatively be used to assess potential flexibility.
For SEC–SAS data a plot of *I*(0) and *R*_g_ as a function of measurement time or measurement frame, and correlated UV traces if used for estimating *C*, including the leading and trailing edge of elution peaks. An *I*(0)/*A*_280_ or *I*(0)/*C* plot as a function of time is also useful. For more complex cases, deconvolution of multiple species in the SEC profile may be needed, for example using the HPLC–SAXS module of *US-SUMO* (http://www.somo.uthscsa.edu/).
Description of the data processing used to obtain the final data set for analysis and modelling [including data reduction to *I*(*q*) *versus q*, solvent subtraction, merging of multiple data sets, extrapolation to infinite dilution *etc.*]. For merged or extrapolated data sets, the original measurements should be available along with the precise protocol used for processing.
For contrast-variation experiments the nature and number of contrast points with a plot of normalized ± [*I*(0)/*C*]^1/2^ *versus* solvent scattering density identifying the total particle solvent match point along with transmissions at each contrast with controls for pure ^1^H_2_O and ^2^H_2_O for calibration.
For contrast-variation experiments on assemblies of components with different mean scattering densities, the *M* or *V* estimates from *I*(0) for each contrast point, Stuhrmann plots and derived *R*_g_ values for individual components and whole particle at infinite contrast and extracted component scattering functions (including cross-term) are all desirable.
Software used for data processing and analysis [*e.g.* *R*_g_, *V*_P_ and *P*(*r*)] including version numbers.

**Table 4:** Summary of reporting guidelines for structure modelling

All software, including version numbers, used for modelling; three-dimensional shape, bead or atomistic modelling.
All modelling assumptions clearly stated, including adjustable parameter values. In the case of imposed symmetry, especially in the case of shape models, comparison with results obtained in the absence of symmetry restraints.
For atomistic modelling, a description of how the starting models were obtained (*e.g.* crystal or NMR structure of a domain, homology model *etc.*), connectivity or distance restraints used and flexible regions specified and the basis for their selection.
Any additional experimental or bioinformatics-based evidence supporting modelling assumptions and therefore enabling modelling restraints or independent model validation.
For three-dimensional models, values for adjustable parameters, constant adjustments to intensity, χ^2^ and associated *P*-values and a clear representation of the model fit to the experimental *I*(*q*) *versus q* including a residual plot that clearly identifies systematic deviations.
Analysis of the ambiguity and precision of models, *e.g.* based on cluster analysis of results from multiple independent optimizations of the model against the SAS profile or profiles, with examples of any distinct clusters in addition to any final averaged model.

**Table:** (*a*) Sample details.

	GI (tetramer)	BSA	CaM
Organism	*Streptomyces rubiginosus*	*Bos taurus*	*Xenopus laevis*
Source (catalogue No. or reference)	Hampton Research (HR7-100)	Sigma–Aldrich (A3294)	*E. coli* expressed (Michie *et al.*, 2016 ▸)
UniProt sequence ID (residues in construct)	P24300 (2–388)	P02769 (25–607)	P62155 (2–149)
Extinction coefficient [*A*_280_, 0.1%(*w*/*v*)]	1.075	0.646	0.178
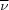 from chemical composition (cm^3^ g^−1^)	0.732	0.732	0.716
Particle contrast from sequence and solvent constituents, 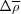 (ρ_protein_ − ρ_solvent_; 10^10^ cm^−2^)	2.87 (12.39 − 9.52)	2.86 (12.38 − 5.92)	3.09 (12.61 − 5.92)
*M* from chemical composition (Da)	172912	66400	16842
SEC–SAXS column, 5 × 150 mm Superdex S200			
Loading concentration (mg ml^−1^)	6	25	20.2
Injection volume (µl)	30	35	35
Flow rate (ml min^−1^)	0.45	0.45	0.45
Average *C* in combined data frames (mg ml^−1^)	0.58 (0.20–1.09)	1.81 (1.01–2.45)	3.09 (2.38–3.55)
Solvent (solvent blanks taken from SEC flowthrough prior to elution of protein)	25 m*M* MOPS, 250 m*M* NaCl, 50 m*M* KCl, 2 m*M* TCEP, 0.1% NaN_3_ pH 7.5		

**Table:** (*b*) SAXS data-collection parameters.

Instrument/data processing	Australian Synchrotron SAXS/WAXS beamline with Dectris PILATUS 1M detector (Kirby *et al.*, 2013 ▸)
Wavelength (Å)	1.0332
Beam size (µm)	250 × 130
Camera length (m)	2.683
*q* measurement range (Å^−1^)	0.00663–0.3104
Absolute scaling method	Comparison with scattering from 1 mm pure H_2_O
Normalization	To transmitted intensity by beam-stop counter
Monitoring for radiation damage	X-ray dose maintained below 210 Gy, data frame-by-frame comparison
Exposure time	Continuous 1 s data-frame measurements of SEC elution
Sample configuration	SEC–SAXS with sheath-flow cell (Kirby *et al.*, 2016 ▸), effective sample path length 0.49 mm
Sample temperature (°C)	22

**Table:** (*c*) Software employed for SAXS data reduction, analysis and interpretation.

SAXS data reduction	*I*(*q*) *versus q* using *ScatterBrain* 2.82 (http://www.synchrotron.org.au/aussyncbeamlines/saxswaxs/software-saxswaxs), solvent subtraction using *PRIMUSqt* (*ATSAS* 2.8.0; Petoukhov *et al.*, 2012 ▸)
Extinction coefficient estimate	*ProtParam* (Gasteiger *et al.*, 2005 ▸)
Calculation of Δ 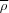 and 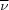 values	*MULCh* 1.1 (06/10/16; Whitten *et al.*, 2008 ▸)
Basic analyses: Guinier, *P*(*r*), *V*_P_	*PRIMUSqt* from *ATSAS* 2.8.0 (Petoukhov *et al.*, 2012 ▸)
Shape/bead modelling	*DAMMIF* (Franke & Svergun, 2009 ▸) and *DAMMIN* (Svergun, 1999 ▸) *via* *ATSAS* online (https://www.embl-hamburg.de/biosaxs/atsas-online/)
Atomic structure modelling	*FoXS* (Schneidman-Duhovny *et al.*, 2013 ▸) *via* web server (https://modbase.compbio.ucsf.edu/foxs/)
	*CRYSOL* from *PRIMUSqt* in *ATSAS* 2.8.1 (Svergun *et al.*, 1995 ▸)
	*MultiFoXS* (Schneidman-Duhovny *et al.*, 2016 ▸) *via* web server (https://modbase.compbio.ucsf.edu/multifoxs/)
	*EOM* (Bernadó *et al.*, 2007 ▸) *via* *ATSAS* online (https://www.embl-hamburg.de/biosaxs/atsas-online/)
Missing sequence modelling	*MODELLER* (https://salilab.org?modeller/; Webb & Sali, 2014 ▸)
Three-dimensional graphic model representations	*PyMOL* v.1.70.0.5 Win64

**Table:** (*d*) Structural parameters.

	GI (tetramer)	BSA	CaM
Guinier analysis			
*I*(0) (cm^−1^)	0.0759 ± 0.0008	0.0861 ± 0.0008	0.0554 ± 0.00008
*R*_g_ (Å)	32.87 ± 0.13	28.33 ± 0.05	21.74 ± 0.06
*q*_min_ (Å^−1^)	0.007	0.007	0.007
*qR*_g_ max (*q*_min_ = 0.0066 Å^−1^)	1.3	1.3	1.3
Coefficient of correlation, *R* ^2^	0.999	0.999	0.999
*M* from *I*(0) (ratio to predicted)	178312 (1.03)	65589 (0.99)	21944 (1.31)
*P*(*r*) analysis			
*I*(0) (cm^−1^)	0.0748 ± 0.00008	0.0850 ± 0.00006	0.0533 ± 0.00006
*R*_g_ (Å)	32.65 ± 0.04	28.32 ± 0.03	22.2 ± 0.06
*d*_max_ (Å)	92	87	72
*q* range (Å^−1^)	0.007–0.243	0.007–0.282	0.0074–0.310
χ^2^ (total estimate from *GNOM*)	0.929 (0.94)	0.858 (0.96)	0.855 (0.91)
*M* from *I*(0) (ratio to predicted value)	180191 (1.04)	65354 (1.00)	21718 (1.29)
Porod volume (Å^−3^) (ratio *V*_P_/calculated *M)*	229000 (1.3)	101000 (1.5)	25200 (1.5)
*V*, *M* using the Fischer method (ratio of *M* to expected)	192400, 157.9 (0.91)	82440, 67.9 (1.02)	21550, 17.7 (1.05)

**Table:** (*e*) Shape model-fitting results.

	GI (tetramer)	BSA	CaM
*DAMMIF* (default parameters, 20 calculations)			
*q* range for fitting (Å^−1^)	0.007–0.243	0.007–0.282	0.007–0.310
Symmetry, anisotropy assumptions	*P*1, none	*P*1, none	*P*1, prolate
NSD (standard deviation), No. of clusters	0.62 (0.01), 1	0.75 (0.63), 6	0.77 (0.02), 4
χ^2^ range	2.25–2.29	0.96–0.99	1.30–1.37
Constant adjustment to intensities	Skipped, unable to determine	1.51 × 10^−4^	1.48 × 10^−4^
Resolution (from *SASRES*) (Å)	37 ± 3	32 ± 3	30 ± 3
*M* estimate as 0.5 × volume of models (Da) (ratio to expected)	134000 (0.77)	66700 (1.00)	16300 (0.97)
*DAMMIN* (default parameters)			
*q* range for fitting (Å^−1^)	0.007–0.243	0.007–0.282	0.007–0.310
Symmetry, anisotropy assumptions	*P*1	*P*1	*P*1
χ^2^, *CORMAP* *P*-values	0.95, 0.04	0.85, 0.16	0.844, 0.53
Constant adjustment to intensities	2.697 × 10^−5^	7.736 × 10^−5^	1.877 × 10^−4^

**Table:** (*f*) Atomistic modelling.

Crystal structures	PDB entry 1oad	PDB entry 4f5s (chain *A*)	PDB entry 1cll+†
*q* range for all modelling	0.007–0.243	0.007–0.282	0.007–0.310
*FoXS* ‡			
χ^2^, *P*-value	1.02, 0.05	4.4, 0.00	9.2, 0.00
Predicted *R*_g_ (Å)	31.70	26.75	21.58
*c*_1_, *c*_2_	1.03, 0.81	0.99, 2.39	0.99, 2.94
*CRYSOL* § (with default parameters)			
No constant subtraction			
χ^2^, *P*-value	1.00, 0.05	2.78, 0.00	15.95, 0.00
Predicted *R*_g_ (Å)	32.69	27.89	22.51
Vol (Å), Ra (Å), Dro (e Å^−3^)	230987, 1.80, 0.0130	76791, 1.80, 0.035	20271, 1.40, 0.025
Constant subtraction allowed			
χ^2^, *P*-value	1.01, 0.05	2.14, 0.00	12.62, 0.00
Predicted *R*_g_ (Å)	32.71	28.01	22.11
Vol (Å), Ra (Å), Dro (e Å^−3^)	226689, 1.40, 0.013	76791, 1.80, 0.037	22012, 1.40, 0.055
Multistate/ensemble models			
Starting crystal structures		PDB entry 4f5s (chain *A*)	PDB entry 1cll+†
Flexible residues		183–187 and 381–384	1–3 (ADQ), 77–87 (KDTDS)
*MultiFoXS* ¶ (10 000 models in starting set)			
No. of states		1	1
χ^2^, *CORMAP* *P*-values		1.05, 0.02	0.85, 0.31
*c*_1_, *c*_2_		0.99, 0.63	1.05, 0.99
*R*_g_ values of each state (Å)		27.59	21.03
Weights *w_n_*		1	1
No. of states		2	2
χ^2^, *CORMAP* *P*-values		0.96, 0.09	0.79, 0.79
*c*_1_, *c*_2_		1.02, 1.21	1.02, 1.50
*R*_g_ values of each state (Å)		26.42, 32.35	22.32, 19.47
Weights *w_n_*		0.83, 0.17	0.70, 0.30
No. of states		3	3
χ^2^, *CORMAP P*-values		0.82, 0.17	0.79, 0.79
*c*_1_, *c*_2_		1.02, 0.94	1.02, 1.52
*R_g_* values of each state (Å)		26.42, 30.43, 29.80	22.32, 30.25, 19.00
Weights *w_n_*		0.74, 0.08, 0.08	0.68, 0.13, 0.18
*EOM* (default parameters, 10 000 models in initial ensemble, native-like models, constant subtraction allowed)			
χ^2^, *CORMAP* *P*-values			0.82, 0.79
Constant subtraction			0
No. of representative structures			13

**Table:** (*g*) SASBDB IDs for data and models.

GI	BS	CaM
SASDCK2	SASDCJ3	SASDCQ2

† PDB entry 1cll+ is PDB entry 1cll plus the missing ADQ at the N-terminus and the C-terminal K missing in the crystal structure.

‡ In *FoXS* the adjustable parameters *c*_1_ and *c*_2_ are adjustments for excluded volume and hydration density. *c*_1_ can vary by 5% (0.95–1.05) and the maximum hydration adjustment *c*_2_ of 4.0 corresponds to ∼0.388 e Å^−3^ (compared with bulk solvent density ρ = 0.334 e Å^−3^).

§ In *CRYSOL* the adjustable parameters are excluded volume (Vol in Å^3^), optimal atomic radius (Ra in Å) and Dro (optimal contrast of the hydration shell in e Å^−3^).

¶ In *MultiFoXS* *c*_1_ and *c*_2_ are the same for all states in a set; the scale factor *c* is then optimized for each state and a relative weight *w_n_* for each state *n* is output.
