# Supplementary material for: 2017 publication guidelines for structural modelling of small-angle scattering data from biomolecules in solution: an update
Source: Acta Crystallogr D Struct Biol. 2017 Aug 18;73(Pt 9):710–28. doi: 10.1107/S2059798317011597 (PMC5586245; doi:10.1107/S2059798317011597)
Supplement: Supplementary file 1 [file d-73-00710-sup1.docx]

**[
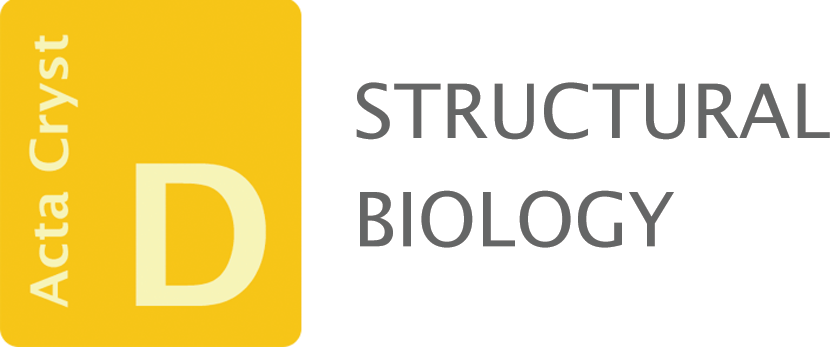
](http://journals.iucr.org/d)**

**Volume 73 (2017)**

**Supporting information for article:**

[**2017 publication guidelines for structural modelling of small-angle scattering data from biomolecules in solution: an update**](http://dx.doi.org/10.1107/S2059798317011597)

**Jill Trewhella, Anthony P. Duff, Dominique Durand, Frank Gabel, J. Mitchell Guss, Wayne A. Hendrickson, Greg L. Hura, David A. Jacques, Nigel M. Kirby, Ann H. Kwan, Javier Pérez, Lois Pollack, Timothy M. Ryan, Andrej Sali, Dina Schneidman-Duhovny, Torsten Schwede, Dmitri I. Svergun, Masaaki Sugiyama, John A. Tainer, Patrice Vachette, John Westbrook and Andrew E. Whitten**

**Supporting Information**

1. Reporting template for tabulating essential SAS data acquisition, sample details, data analysis, modelling fitting and software used.

| (*a*) Sample details | | | | |
| --- | --- | --- | --- | --- |
|  | Sample 1 | | Sample 2 | Sample 3, etc. |
| Organism |  | |  |  |
| Source |  | |  |  |
| Description - sequence (including tags) + bound ligands/modifications, etc. |  | |  |  |
| Extinction coefficient |  | |  |  |
| Partial specific volume |  | |  |  |
| Scattering contrast, solute and solvent scattering lengths |  | |  |  |
| *M* from chemical composition |  | |  |  |
| For SEC-SAS, loading volume/concentration, flow rate |  | |  |  |
| Concentration (range/values) measured and method |  | |  |  |
| *M* from chemical composition |  | |  |  |
| Solvent details |  | |  |  |
| (*b*) SAXS data collection parameters | | | | |
| Source, instrument and description or reference | | | | |
| Wavelength | | | | |
| Beam geometry (size, sample – detector distance) | | | | |
| *q*-measurement range (Å^-1^ or nm^-1^) | | | | |
| Absolute scaling method | | | | |
| Basis for normalization to constant counts | | | | |
| Method for monitoring radiation damage, X-ray dose where relevant | | | | |
| Exposure time, number of exposures | | | | |
| Sample configuration including path length and flow rate where relevant | | | | |
| Sample temperature | | | | |
| (*c*) Software employed for SAXS data reduction, analysis and interpretation | | | | |
| SAXS data reduction to (sample – solvent), extrapolation, merging, desmearing etc. as relevant | | | | |
| Calculation of extinction coefficient from sequence | | | | |
| Calculation of *Δ*$\bar{\rho}$ and $\bar{\vartheta}$ values from chemical composition | | | | |
| Basic analyses: Guinier, *P*(*r*), Porod volume, volume of correlation | | | | |
| Shape/bead modelling | | | | |
| Atomic structure modelling (homology, rigid body, ensemble) | | | | |
| 3D graphic model representations | | | | |
| (*d*) Structural parameters | | | | |
| Guinier Analysis | Sample 1 | Sample 2 | | Sample 3, etc. |
| *I(0)* |  |  | |  |
| *R_g_* |  |  | |  |
| *qR_g_* range |  |  | |  |
| Quality-of-fit parameter (with definition) |  |  | |  |
| *M* from *I*(0) (ratio to expected value) |  |  | |  |
| *P*(*r*) analysis | Sample 1 | Sample 2 | | Sample 3, etc. |
| *I(0)* |  |  | |  |
| *R_g_* |  |  | |  |
| *d_max_* |  |  | |  |
| *q* range |  |  | |  |
| Quality-of-fit parameter (with definition) |  |  | |  |
| *M* from *I*(0) (ratio to expected value) |  |  | |  |
| *V_P_* and/or *V_c_* |  | |  |  |
| (*e*) Shape modelling results (a complete panel for each method) | | | | |
|  | Sample 1 | Sample 2 | | Sample 3, etc. |
| *q* range for fitting |  |  | |  |
| Symmetry/anisotropy assumptions |  |  | |  |
| Ambiguity measure(s) with definitions |  |  | |  |
| χ^2^ range value/range, other quality of fit parameters |  |  | |  |
| Adjustable parameters in the model fit |  |  | |  |
| Model volume and/or *M_r_* estimate |  |  | |  |
| Model precision/resolution |  |  | |  |
| For multiple phase shape models, *R_g_* values and relative phase volumes for multiple phase shape models |  |  | |  |
| (*f*) Atomistic modelling | | | | |
|  | Sample 1 | | Sample 2 | Sample 3, etc. |
| *q* range for fitting |  | |  |  |
| Symmetry assumptions |  | |  |  |
| Any masures of model precision |  | |  |  |
| χ^2^ range value/range, other quality of fit parameters |  | |  |  |
| Adjustable parameters in the model fit |  | |  |  |
| Domain/subunit contacts and regions of presumed flexibility for atomistic modelling |  | |  |  |
| (*g*) Data and model deposition IDs |  | |  |  |

**
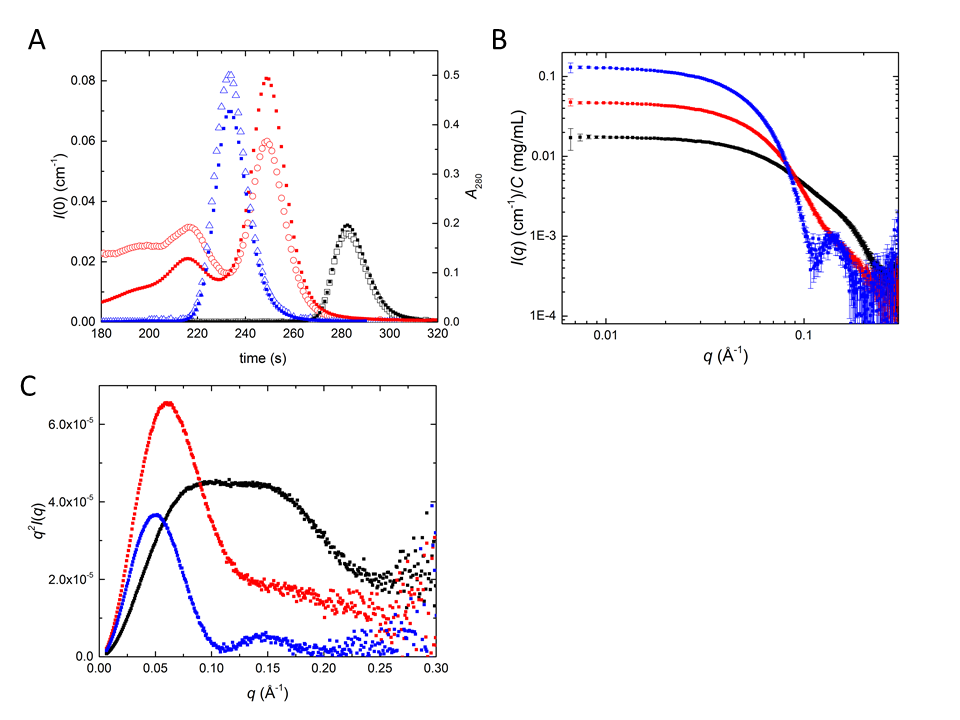
**

1. **A.** Overlaid plots of *I*(0) (filled symbols) and A280 (hollow symbols) as a function of time/measurement frame showing the good correspondence in peak shape that facilitates concentration estimates for a set of 1 second measurement frames. These plots are raw values and have not been corrected for the shortened pathlengths for the shear-flow cell of UV cell. **B.** log*I*(*q*) v log*q* plots showing the expected near zero slope at low-*q* expected for monodisperse scattering particles of similar size. **C.** Kratky plots for GI, BSA, and CaM. The rising Kratky plot for *q* values > 0.25 Å^-1^ for BSA and CaM are indicative of flexibility in these proteins. Color key is as in main figures: GI (blue), CaM (black) and BSA (red).
